# Unlocking the therapeutic mechanism of *Caesalpinia sappan*: a comprehensive review of its antioxidant and anti-cancer properties, ethnopharmacology, and phytochemistry

**DOI:** 10.3389/fphar.2024.1514573

**Published:** 2025-01-07

**Authors:** Estéfani Alves Asevedo, Livia Ramos Santiago, Hyo Jeong Kim, Rony Abdi Syahputra, Moon Nyeo Park, Rosy Iara Maciel Azambuja Ribeiro, Bonglee Kim

**Affiliations:** ^1^ Experimental Pathology Laboratory, Midwest Campus, Federal University of São João del-Rei, Divinópolis, Brazil; ^2^ Department of Pathology, College of Korean Medicine, Kyung Hee University, Seoul, Republic of Korea; ^3^ Department of Pharmacology, Faculty of Pharmacy, Universitas Sumatera Utara, Sumatera Utara, Indonesia

**Keywords:** *Caesalpinia sappan*, antioxidant, phytochemistry, pharmacological properties, brazilin, protosappanin B, caesalpanins C

## Abstract

Herbal medicine are an invaluable reservoir of bioactive compounds, offering immense potential for novel drug development to address a wide range of diseases. Among these, *Caesalpinia sappan* has gained recognition for its historical medicinal applications and substantial therapeutic potential. This review explores the ethnopharmacological significance, phytochemical composition, and pharmacological properties of *C. sappan*, with a particular focus on its anticancer activities. Traditionally, *C. sappan* has been utilized for treating respiratory, gastrointestinal, and inflammatory conditions, demonstrating its broad therapeutic scope. The plant’s rich array of bioactive compounds—flavonoids, triterpenoids, phenolic acids, and glycosides—forms the basis of its potent antioxidant, anti-inflammatory, and pharmacological effects. Modern pharmacological research has further substantiated its versatility, revealing anticancer, anti-diabetic, anti-infective, and hepatoprotective properties. However, significant challenges remain, including the need to unravel the precise molecular mechanisms underlying its anticancer effects, refine extraction and isolation methods for bioactive compounds, and validate its safety and efficacy through well-designed clinical trials. Particularly noteworthy is *C. sappan*’s potential in combination therapies, where it may synergistically target multiple cancer pathways, enhance therapeutic outcomes, and mitigate adverse effects. This review synthesizes the findings from the past decade, providing a comprehensive evaluation of C. sappan’s pharmacological promise while identifying critical areas for future research. By addressing these gaps, C. sappan could serve as a cornerstone for innovative therapeutic strategies, offering hope for improved management of cancer and other complex diseases.

## 1 Introduction

Herbal medicine remains a cornerstone in the search for novel drug candidates, offering a wealth of pharmacologically active compounds that have been used for centuries in traditional medicine (He et al., 2024). Notably, approximately 25% of drugs currently utilized in clinical practice are derived from plant-based compounds, underscoring the significance of the plant kingdom as a valuable source for drug discovery (Haque et al., 2024). Among these botanical resources, *Caesalpinia sappan* has garnered significant attention for its dual role as a traditional medicinal remedy and a natural food coloring agent, with its use dating back to ancient times (Sasarom et al., 2024). C. sappan is rich in diverse secondary metabolites, including flavonoids, saponins, alkaloids, tannins, and phenolics, which have been extensively studied for their therapeutic potential (Chukiatsiri et al., 2024; Sucita et al., 2024). Among its bioactive constituents, brazilin and brazilein have emerged as the most studied compounds, demonstrating potent cytotoxic effects against various tumor cell lines, as well as chemopreventive properties (Jenie et al., 2023). Brazilin, in particular, exhibits a wide range of pharmacological activities, including antioxidant, anti-inflammatory, antibacterial, and hypoglycemic effects. It has also shown promise in addressing complex diseases such as osteoarthritis, Parkinson’s disease, and Alzheimer’s disease (Sugiaman et al., 2024). Additionally, other compounds like cassane diterpenoids and sapanone A have exhibited anti-inflammatory, antimalarial, antimicrobial, antiviral, antihyperglycemic (Su et al., 2024) antioxidant, and anticancer properties (He et al., 2024). The long history of culinary and medicinal applications of *C. sappan* has inspired a growing body of research aimed at elucidating its pharmacological properties and therapeutic potential. Despite extensive investigations, significant gaps remain in understanding its precise mechanisms of action. To advance the development of *C. sappan*-based therapeutic agents, this review seeks to provide a comprehensive summary of the current knowledge on the pharmacological properties of *C. sappan*, highlighting its potential contributions to drug development and addressing the need for further research into its mechanisms of action.

## 2 Methodology

To gather relevant data for this review, articles related to *C. sappan* published within the last decade were sourced from major academic databases, including PubMed, Scopus, and Web of Science. Search terms were strategically grouped based on descriptors from the DeCS (Health Sciences Descriptors) and Medical Subject Headings (MeSH), with the primary search term being “*C. sappan*.” The inclusion criteria focused on studies exploring both crude extracts and isolated phytoconstituents of *C. sappan* that demonstrated significant pharmacological properties. This review is structured to cover *C. sappan*’s broad therapeutic potential, addressing its antioxidant, anti-inflammatory, anti-infectious, and anticancer properties, along with its effectiveness in treating diseases such as diabetes, cardiovascular conditions, and joint-related disorders.

## 3 Ethnopharmacology

Herbal medicine has been an integral part of traditional clinical treatment for thousands of years in Korea, China, Japan, and other East Asian countries (Koonrungsesomboon et al., 2024). In recent decades, extensive research has focused on the therapeutic potential of natural antioxidants, particularly those derived from medicinal plants. These natural antioxidants are widely distributed in herbal medicines and exhibit a broad spectrum of biological activities, including anti-cancer, anti-inflammatory, and anti-aging effects (Halliwell, 2024).

### 3.1 Ethnopharmacological relevance of *C. sappan*

**FIGURE 1:**
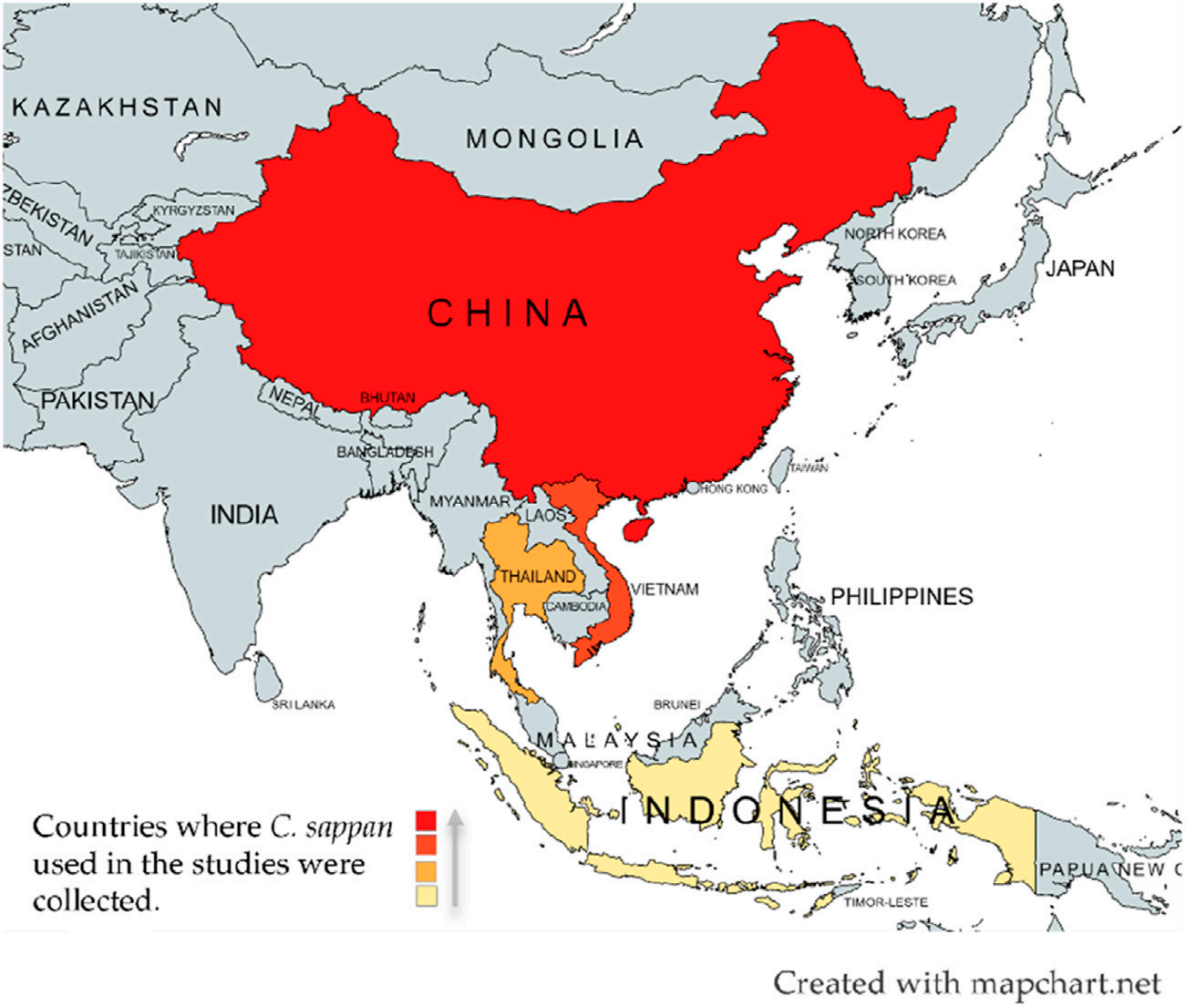
Geographic Locations of *Caesalpinia sappan* Plant Samples Used in Studies Reviewed. Most of the *C. sappan* plants mentioned were harvested in China, followed by Vietnam, Thailand, and Indonesia.

*Caesalpinia sappan*, a plant with deep-rooted ethnopharmacological significance, has been traditionally utilized in regions such as India, Myanmar, Vietnam, Sri Lanka, and the Malay Peninsula, and is also found in China, especially in provinces like Yunnan, Guizhou, Sichuan, Guangdong, Guangxi, Fujian, and Taiwan (Figure 1) (Wang, Sun and Zhou, 2011). In traditional Chinese medicine (TCM) and other Asian medical systems, *C. sappan* has been widely valued for its therapeutic versatility. For instance, in TCM, it has been employed as an analgesic and anti-inflammatory agent to address a variety of conditions such as white blood cell disorders, complications of diabetes, leprosy, skin diseases, and gynecological disorders (Liang et al., 2013; Widodo et al., 2022). In India and other parts of Southeast Asia, it has been used to improve blood circulation and alleviate ailments like sprains, convulsions, and diabetic complications, reflecting its significance in Ayurvedic and other indigenous medicinal systems (Wang et al., 2022; Wu et al., 2022). Notably, its heartwood has been used in numerous traditional formulations for treating skin conditions and promoting overall health, indicating its broad acceptance across diverse cultural practices.

### 3.2 Traditional formulations and historical uses

Ethnopharmacologically, *C. sappan* is incorporated into traditional herbal formulations such as Hua-Zheng-Hui-Sheng-Dan and Sa-Tri-Lhung-Klod, which are used in Chinese and Thai medicine, respectively, for their anti-inflammatory and anti-cancer effects. These formulations are designed to synergistically enhance therapeutic outcomes, targeting multiple pathways, and are often utilized in managing chronic and inflammatory conditions. Additionally, brazilin, a notable compound isolated from the heartwood, was historically used as a natural red dye across China, Japan, and India, underscoring its cultural and economic value (Nathan and Rani, 2021).

### 3.3 Botanical and pharmacological insights

Belonging to the family Fabaceae, *C. sappan* is a small to medium-sized tree, reaching up to 10 m in height with a trunk diameter of approximately 14 cm and alternate bipinnate leaves (Mariappan et al., 2014). Its dried heartwood has shown remarkable pharmacological properties, including antioxidant, antibacterial, and anti-cancer activities, which align with its historical uses. Research indicates its active constituents, such as brazilin and brazilein, play pivotal roles in its therapeutic efficacy. These compounds have demonstrated promising anti-cancer properties *in vitro* and *in vivo*, highlighting their potential for modern drug development. Recent research underscores *C. sappan*’s pharmacological versatility. Its extracts and isolated compounds exhibit wide-ranging biological effects, including antioxidant, anti-inflammatory, antimicrobial, and anticancer activities (Vij et al., 2023). In cancer therapy, there is increasing interest in alternative treatments that offer efficacy with fewer side effects compared to conventional first-line therapies. Given the growing recognition of the role phytoconstituents play in this search, compounds isolated from *C. sappan* heartwood and seeds have shown notable selectivity against a variety of cancer cell lines, predominantly through the activation of apoptotic pathways (Bao et al., 2016; Kumar et al., 2024; Seo et al., 2020). Furthermore, some constituents have demonstrated anti-neuroinflammatory effects in in vitro models, further supporting the plant’s protective properties, which are largely attributed to its potent antioxidant capacity (Nirmal and Panichayupakaranant, 2015; Pyun et al., 2022; Wang et al., 2023). In addition to cancer, *C. sappan* has shown promise in treating joint-related disorders, cardiovascular diseases, and diabetes, with positive outcomes reported in several studies (Wediasari et al., 2020; Weinmann et al., 2018; Wu et al., 2022). This review aims to consolidate recent research on *C. sappan* from the past decade, highlighting its efficacy across multiple disease models and providing insights into its mechanisms of action. Through this synthesis, we aim to underscore the therapeutic potential of *C. sappan* as a versatile and valuable resource in modern medicine.

## 4 Analysis of key phytochemicals in *C. sappan*

The heartwood of *C. sappan* is the richest source of the plant’s bioactive compounds, although research has also focused on key compounds extracted from its seeds. Phytochemicals from the heartwood are primarily homoisoflavonoids, while those isolated from the seeds are generally classified as diterpenoids. Homoisoflavonoids, which are characterized by the addition of a carbon atom to the skeleton structure of traditional isoflavonoids, are prominent in the Caesalpinia genus. While Sappanin is a common homoisoflavonoid in related species, the rarer brazilin is the key compound of interest in *C. sappan* (Castelli and López, 2017). Compounds isolated from the seeds of *C. sappan* typically belong to the diterpenoid class a diverse group of phytochemicals composed of 20 carbon terpenoids formed from four isoprene units. Interest in diterpenoids surged after the approval of the diterpenoid taxane drug, Taxol (derived from Taxus brevifolia), as a first-line treatment for cancer (Yarnell, 2007). The discovery of diterpenoids’ significant biological activity in cancer therapies has expanded interest in their potential medicinal applications, making *C. sappan* a subject of growing pharmacological interest. This unique combination of bioactive compounds from both heartwood and seeds underlines *C. sappan*’s versatility and potential for developing novel therapeutic agents across a variety of medical fields. Contributing to this, the toxicity prediction of the *C. sappan* constituents presented in Table 1 indicates that they mostly have a toxicity class between 4 and 5, with emphasis on Sappanone A with a predicted LD_50_ of 3,800 mg/kg, and lowest predicted toxicity.

**TABLE 1:** Chemical structure and toxicity of C. sappan compounds.

Compound	Chemical structure	LD_50_* (mg/kg)	Toxicity* class	Efficacy	References
Brazilein	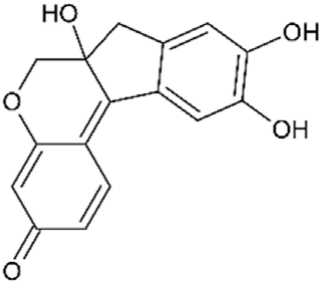	2,000	4	Anticancer Anti-inflammatory Antibacterial	Handayani et al. (2017), Handayani et al. (2022), Kim, et al. (2015a), Kwak et al. (2021), Wudtiwai et al. (2023), Zuo et al. (2014)
Brazilin	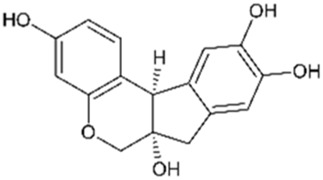	800	4	Anticancer Antioxidant Anti-inflammatory Antibacterial Antiviral	Mueller et al. (2016), Nirmal and Panichayupakaranant (2015), Tewtrakul et al. (2015), Uddin et al. (2015), Wang et al. (2019), Zhang et al. (2014), Chatterjee et al. (2022), Handayani et al. (2017), Jenie et al. (2018), Jeon et al. (2014), Kang et al. (2018), Kim et al. (2015b), Suyatmi et al. (2022), Zhang et al. (2014)
Deoxysappanone B	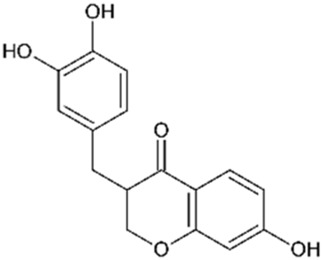	2,000	4	Anti-neuroinflammatory	Zeng et al. (2015)
Episappanol	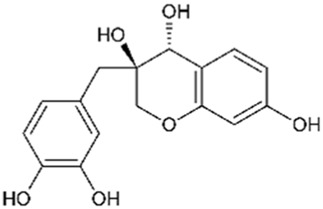	1,190	4	Anti-inflammatory	Mueller et al. (2016)
Phanginin A	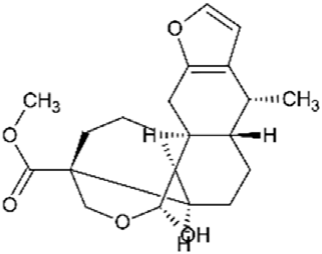	244	3	Cytotoxic effect	Tran et al. (2015)
Protosappanin A	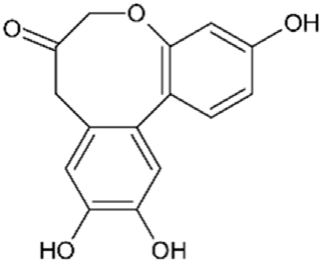	777	4	Anti-neuroinflammatory Antibacterial	Wang et al. (2017)
Protosappanin B	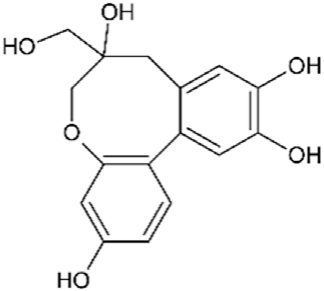	777	4	Anti-inflammatory Antibacterial	Mueller et al. (2016)
Sappanol	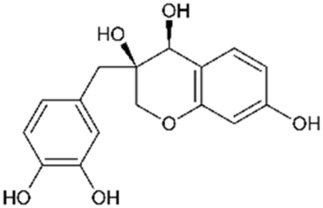	2,500	5	Antioxidant Anti-inflammatory	Mueller et al. (2016), Uddin et al. (2015)
Sappanone A	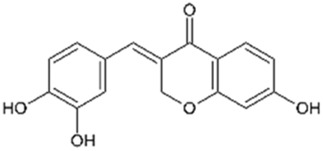	3,800	5	Antioxidant Anti-inflammatory Antibacterial	Lee et al. (2015a), Liu et al. (2016), Wang et al. (2023)
Sappanchalcone	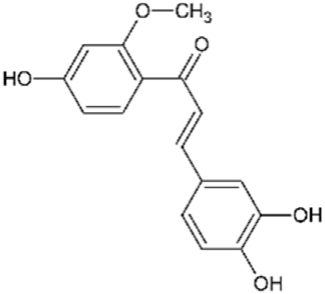	3,000	5	Anticancer	Zhang et al. (2014)
3-Deoxysappanchalcone	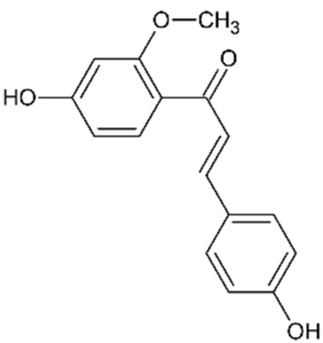	3,000	5	Anticancer Anti-inflammatory Antibacterial	Kim et al. (2014), Kwak et al. (2021), Zhao et al. (2019)

*Values predicted by Tox prediction on website *ProTox 3.0* (https://tox.charite.de/protox3/index.php?site=compound_input).

**FIGURE 2:**
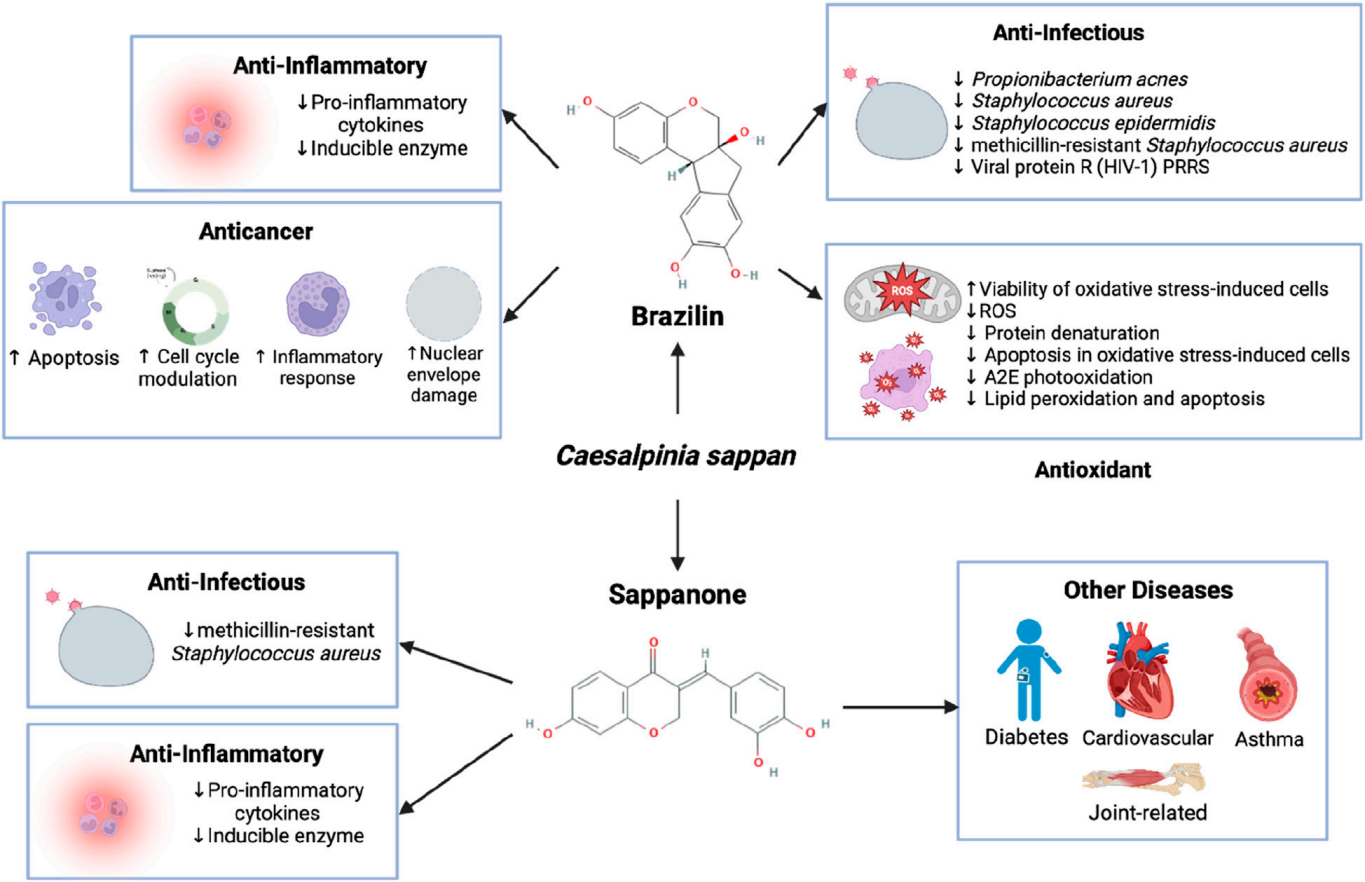
Antioxidant, anti-infectious, anti-inflammatory, and anticancer Properties of brazilin and sappanone. Sappanone has also been the subject of numerous investigations due to its beneficial health properties, which justify its frequent study. The compound exhibited anti-infectious effect against drug-resistant bacteria, reduced pro-inflammatory cytokines in asthma *in* an *in vitro* model, and showed efficacy in treating various diseases. Most of sappanone’s effects where was related to the activation of anti-inflammatory mechanisms.

Among the studies highlighted in this review, the heartwood of *C. sappan* was the most frequently utilized source, with Brazilin emerging as the most extensively researched compound. Brazilin demonstrated potent cytotoxic effects across a wide range of cancer cell lines, while also exhibiting remarkable antioxidant properties. It was particularly effective in shielding non-cancerous cells from oxidative stress, thereby preventing protein denaturation. Beyond its anticancer and antioxidant capacities, Brazilin also displayed significant anti-infectious properties. It was found to combat both common and drug-resistant bacterial strains, as well as viruses, by effectively reducing viral protein R activity. These findings underscore Brazilin’s multifaceted potential as a therapeutic agent with broad-spectrum applications in cancer treatment, infection control, and cellular protection. Sappanone has also been widely investigated in the last decade, showing efficacy as an anti-inflammatory agent, capable of overcoming bacterial resistance and mainly having an effect against different diseases, such as diabetes, cardiovascular disease, asthma and even joint-related disease (Figure 2).

## 5 Antioxidant and anti-inflammatory research trend

**FIGURE 3:**
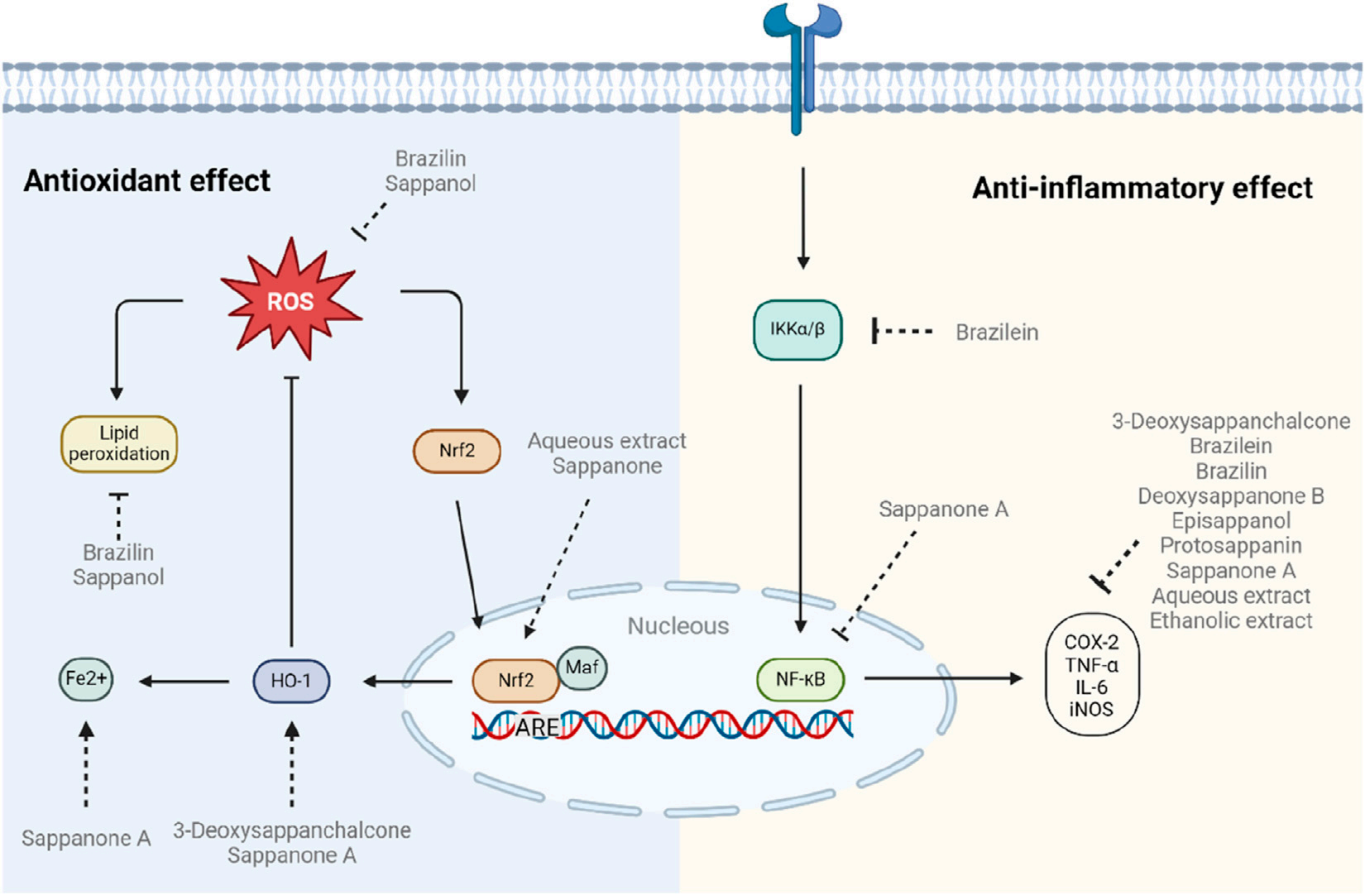
Antioxidant and anti-inflammatory effect of *C. sappan* Extracts and Isolated Compounds. Regarding antioxidant effect, Brazilin and Sappanol reduced ROS and lipid peroxidation, while Aqueous Extract of *C. sappan* and Sappanone upregulated Nrf2, 3-Deoxysappanchalcone and Sappanone A increased HO-1 activity, and Sappanone favored Fe^2+^ release. In relation to anti-inflammatory effects, many compounds and extracts such as, 3-Deoxysappanchalcone, Brazilein, Brazilin, Deoxysappanone B, Episappanol, Protosappanin, Sappanone A, Aqueous Extract, and Ethanolic Extract downregulated pro-inflammatory enzymes and cytokines, brazilein inhibited the key pro-inflammatory protein IKK, and Sappanone A downregulated NF-κB.

Numerous studies have highlighted the potent antioxidant and anti-inflammatory properties of various compounds isolated from *C. sappan*, solidifying its role as a promising therapeutic agent, the modulation of these signaling pathways is summarized in Figure 3. Table 2 presents key studies that explored these activities and the underlying mechanisms of action of the isolated compounds. Kim et al. (2014) demonstrated the anti-inflammatory effects of 3-deoxysappanchalcone, a chalcone derived from *C. sappan* heartwood. This compound exhibited its therapeutic potential by inducing heme oxygenase-1 (HO-1) expression and activating the AKT/mTOR pathway, leading to the inhibition of NO and IL-6 production in LPS-stimulated RAW264.7 cells (Kim et al., 2014). In the same vein, Kim K. J. et al. (2015) also investigated brazilein and found it to effectively suppress inflammatory mediators. By downregulating iNOX and COX2 expression, brazilein reduced pro-inflammatory cytokines and inhibited NF-κB luciferase activity (Kim K. J. et al., 2015). Tewtrakul et al. (2015) identified brazilin as the most potent compound from *C. sappan* roots, significantly inhibiting NO production in LPS-induced RAW264.7 cells. Sappanchalcone also displayed notable efficacy. Their findings revealed that brazilin also inhibited PGE2 and TNF-α production, suggesting a mechanism involving the downregulation of iNOS, COX-2, and TNF-α (Tewtrakul et al., 2015). In another study, Uddin et al. (2015) found that sappanol and brazilin provided protection against oxidative stress in H₂O₂-induced RGC-5 cells. These compounds effectively reduced A2E photooxidation and lipid peroxidation, improving cell viability while decreasing apoptosis and ROS generation (Uddin et al., 2015). Mueller et al. (2016) isolated five fractions from ethanolic extracts of *C. sappan* heartwood—episappanol, protosappanin C, brazilin, (iso-) protosappanin B, and sappanol—and evaluated their anti-inflammatory activities. All five compounds inhibited IL-6 and TNF-α secretion in both LPS-stimulated RAW 264.7 cells and IL-1β-stimulated SW1353 cells. Additionally, sappanol enhanced IL-10 secretion, with brazilin showing the strongest anti-inflammatory activity (Mueller et al., 2016). Nirmal and Panichayupakaranant further substantiated the versatile properties of brazilin, demonstrating its antioxidant, antibacterial, and anti-inflammatory effects. Their study revealed brazilin’s ability to perform radical scavenging and prevent denaturation, thereby supporting its role in disease mitigation (Nirmal and Panichayupakaranant, 2015). Wang et al. (2019) provided insights into the neuroprotective role of brazilin, demonstrating its antidepressant and anxiolytic effects in models of H₂O₂-induced oxidative injury in PC12 cells and CMS-induced depression in mice (Wang et al., 2019). Similarly, Zeng et al. (2015) explored the anti-neuroinflammatory potential of Deoxysappanone B, reporting its ability to inhibit NO, PGE₂, TNF-α, IL-6, and ROS production in LPS-induced BV-2 microglia and microglia-neuron co-cultures (Zeng et al., 2015). Wang et al. (2017) highlighted Protosappanin A as an effective anti-neuroinflammatory agent. By inhibiting the production of TNF-α, IL-1β, and suppressing the JAK2/STAT3 pathway, Protosappanin A exhibited strong anti-inflammatory properties in LPS-stimulated BV2 cells (Wang et al., 2017). In their search for natural treatments for chronic obstructive pulmonary disease (COPD), Wang et al. (2023) identified Sappanone A through molecular docking and dynamics analysis as a potent phosphodiesterase 4 (PDE4) inhibitor. *In vitro* and *in vivo* studies confirmed its efficacy, with Sappanone A decreasing TNF-α levels, scavenging DPPH radicals, and reducing inflammation in bronchoalveolar lavage fluid (Wang et al., 2023). Lee H. et al. (2015) further corroborated Sappanone A’s anti-inflammatory effects through modulation of the Nrf2/NF-κB pathway. Sappanone A significantly downregulated pro-inflammatory mediators in LPS-stimulated RAW264.7 cells and provided protection against LPS-induced mortality in mice (Lee H. et al., 2015). Liu et al. (2016) examined the effectiveness of Sappanone A in an asthma model, noting reductions in IL-4, IL-5, IL-13, and OVA-specific IgE levels in bronchoalveolar lavage fluid. The treatment also upregulated IFN-γ, reducing airway inflammation and mucus hypersecretion by activating the Nrf2 pathway (Liu et al., 2016).

**TABLE 2:** Antioxidant and anti-inflammatory effect of *C. sappan* isolated compounds.

Compound/extract	Experimental model	Dose/Duration	Efficacy	Mechanism	References
3-Deoxysappanchalcone	RAW264.7 cells	1, 3, 10, 30 μM 24 h	Antioxidant Anti-inflammatory	↑ HO-1, p-mTOR, 4E-BP1, S6K1, AKT ↓ NO, IL-6 AKT, mTOR	Kim et al. (2014)
Brazilein	RAW264.7 cells	10, 30, 50 μM 24 h	Anti-inflammatory	↓ iNOS, COX2, IL-1β, MCP-1, MIP-2, IL-6, p-JNK, p-ERK, p-p38MAPK, p-IKKα/β, IRAK4	Kim, et al. (2015a)
Brazilin	RAW264.7 cells	3, 10, 30, 100 μM 20 h	Anti-inflammatory	↓ iNOS, COX-2, TNF-α	Tewtrakul et al. (2015)
Brazilin	H₂O₂-RGC-5 cells	1, 10, 20, 50 μM 24 h	Antioxidant Retinal protection	↓ A2E photooxidation, lipid peroxidation, apoptosis, ROS	Uddin et al. (2015)
Brazilin	RAW 264.7, SW1353 cells	5, 10, 20, 50 μg/mL 24 h	Anti-inflammatory	↓ IL-6, TNF-α	Mueller et al. (2016)
Brazilin	DPPH radical scavenging, reducing power, β-carotene bleaching, anti-denaturation activity using BSA	1-10, 10-100, 10-100 μg/mL	Antioxidant Anti-inflammatory	↑ reducing power ↓ protein denaturation	Nirmal and Panichayupakaranant (2015)
Brazilin	H₂O₂-PC12 cells/IRC mice	10, 20 µM/10 mg/kg	Antioxidant, Antidepressant, Anxiolytic-Like Effects	↑ Cell viability ↓ Apoptosis	Wang et al. (2019)
Deoxysappanone B	co-culture system BV2 microglia w/neuron	10, 20, 50 μM 1, 24 h	Antioxidant Anti-neuroinflammatory	↓ ROS, IL-6, TNF-α, NF-кB, IKK, p-ERK, p-p38	Zeng et al. (2015)
	Balb/c mice	10 mg/kg 1 h after LPS (1 mg/kg) 3 h		↓ iNOS, Il-6, Il-1β	
Episappanol	RAW 264.7, SW1353 cells	5, 10, 20, 50 μg/mL 24 h	Anti-inflammatory	↓ IL-6, TNF-α	Mueller et al. (2016)
Protosappanin A	BV2 microglia	12.5, 25, 50 μM 4, 24 h	Anti-neuroinflammatory	↓ NO, TNF-α, IL-1β, IL-6, MCP-1, p-JAK2, p-STAT3	Wang et al. (2017)
Protosappanin C Protosappanin B	RAW 264.7, SW1353 cells	5, 10, 20, 50 μg/mL 24 h	Anti-inflammatory	↓ IL-6, TNF-α	Mueller et al. (2016)
Sappanol	H₂O₂-RGC-5 cells	1, 10, 20, 50 μM 24 h	Antioxidant Retinal protection	↓ A2E photooxidation, lipid peroxidation, apoptosis, ROS	Uddin et al. (2015)
Sappanol	RAW 264.7, SW1353 cells	5, 10, 20, 50 μg/mL 24 h	Anti-inflammatory	↑ IL-10	Mueller et al. (2016)
Sappanone A	Molecular dynamics simulation	—	Antioxidant Anti-inflammatory	↓ PDE4 HAT mechanism	Wang et al. (2023)
	RAW264.7 cells	10 μM		↓ TNF-α, MDA ↑ Fe^2+^	
	ICR mice	50 mg/kg/day and 100 mg/kg/day 7 days		↓ TNF-α	
Sappanone A	RAW264.7 cells	5, 15, 30 μM 24 h	Antioxidant Anti-inflammatory	↓ NO, PGE2, IL-6, iNOS, COX-2, NF-κB ↑ HO-1, Nrf2	Lee et al. (2015a)
Sappanone A	BALB/c mice	12.5, 25 and 50 mg/kg 24 h	Antioxidant Anti-inflammatory (asthma)	↑ IFN-γ, Nrf2, HO-1 ↓ IL-4, IL-5, IL-13	Liu et al. (2016)

Abbreviation: ↓, downregulation; ↑,upregulation; p-, phosphorylated; HO-1, heme oxigenase-1; mTOR, mammalian target of rapamycin; 4E-BP1, eukaryotic translation initiation factor 4E-binding protein 1; S6K1, S6 kinase; AKT, protein kinase B; NO, nitric oxide; IL-6, pro-inflammatory cytokines interleukin 6; iNOS, inducible nitric oxide synthase; COX-2, cyclooxygenase-2; IL-1β, pro-inflammatory cytokines interleukin 1β; MCP-1, monocyte chemoattractant protein-1; MIP-2, macrophage inflammatory proteins-2; JNK, c-Jun N-terminal kinase; ERK, extracellular signal-regulated kinase; p38MAPK, p38 mitogen-activated protein kinase; IKKα/β, inhibitory kappa B kinase alpha/beta; IRAK4, interleukin-1 receptor-associated kinase 4; TNF-α, tumor necrosis factor alpha; IL-10, pro-inflammatory cytokines interleukin 10; ROS, reactive oxygen species; NF-кB, nuclear factor kappa B; IKK, IkappaB kinase; JAK2, Janus Quinase 2; STAT3, signal transducer and activator of transcription 3; PDE4, phosphodiesterase isozyme 4; HAT, histone acetyltransferase; MDA, malondialdehyde; PGE2, prostaglandin E2; Nrf2, nuclear factor erythroid 2-related factor 2; IFN-γ, interferon-gamma; IL-4, pro-inflammatory cytokines interleukin 4; IL-5, pro-inflammatory cytokines interleukin 5; IL-13, pro-inflammatory cytokines interleukin 13.

Table 3 presents a summary of studies directly investigating the antioxidant and anti-inflammatory properties of *C. sappan* extracts. Pyun et al. (2022) explored the therapeutic effects of aqueous *C. sappan* heartwood extract on inflamed nasal epithelial cells and an allergic rhinitis model in mice. Their findings demonstrated a significant reduction in ROS production and inhibition of inflammatory mediators induced by IL-4/IL-13 in primary human nasal epithelial cells. The underlying mechanisms involved regulation of the ERK-MAPK and Nrf2/HO-1 signaling pathways, indicating a strong anti-inflammatory and antioxidant response (Pyun et al., 2022). Chen and Zhang (2014) conducted an extensive evaluation of 81 Chinese herbs, including *C. sappan*, on their ability to modulate inducible nitric oxide synthase (iNOS) activity in LPS/IFNγ-co-stimulated RAW264.7 cells. They reported that the ethanolic extract of C. sappan significantly suppressed NO production, underscoring its anti-inflammatory potential (Chen and Zhang, 2014). Similarly, Pattananandecha et al. (2022) showed that various ethanolic extracts from *C. sappan* heartwood inhibited NO and iNOS production in RAW264.7 cells wi(th minimal cytotoxicity. In addition, these extracts demonstrated potent inhibition of COX-2 production in HT-29 and LPS/IFN-γ co-stimulated HCT116 cells, reinforcing *C. sappan*’s role as an anti-inflammatory agent (Pattananandecha et al., 2022). Wan et al. (2019) investigated the neuroprotective potential of C. sappan ethanolic extract using a Rat Middle Cerebral Artery Occlusion (MCAO) Model, bioinformatics analysis, and human bone marrow neuroblastoma (SH-SY5Y) and rat pheochromocytoma (PC12) cells. The study revealed that *C. sappan* reversed MCAO-induced cerebral injury, inhibited neuronal apoptosis, and mitigated neuroinflammation by reducing neutrophil infiltration, astroglial activation, collagen deposition, and DNA damage/telomere stress. The extract exerted these effects by activating the JAK-STAT and HSP90 signaling pathways, showcasing its multifaceted neuroprotective and anti-inflammatory actions (Wan et al., 2019).

**TABLE 3:** Antioxidant and anti-inflammatory effect of *C. sappan* extracts.

Compound/extract	Experimental model	Dose/Duration	Efficacy	Mechanism	References
Aqueous extract	Primary human nasal epithelial cells	1, 3, 10 μg/mL	Antioxidant Anti-inflammatory (allergic rhinitis)	↑ HO-1, NQO1, SOD1, Nrf2 (nuclear) ↓ OVA-specific IgE, histamine, IL-5, IL-13, p-ERK, Keap1, Nrf2 (cytosol)	Pyun et al. (2022)
	Balb/c mice	10 mg/kg 1 h		↓ iNOS, IL-6, IL-1β	
Ethanolic extract	RAW264.7 cells	100 μg/mL 24 h	Anti-inflammatory	↓ NO	Chen and Zhang (2014)
Ethanolic extract	RAW264.7, HT-29, HCT116 cells	50 μg/mL	Anti-inflammatory	↓ NO, iNOS, COX-2	Pattananandecha et al. (2022)
Ethanolic extract	MCAO rats	300 mg/kg	Anti-neuroinflammatory	↑ HSP70 ↓ c-caspase3, c-PARP, p-JAK2, p-STAT3, HSP90	Wan et al. (2019)
	PC12, SH-SY5Y cells	2.4 μg/mL			

Abbreviation:↓, downregulation; ↑, upregulation; p-, phosphorylated; c-, cleaved; HO-1, heme oxygenase 1; NQO1, antioxidative enzyme NAD(P)H quinone oxidoreductase 1; SOD1, superoxide dismutase type 1; Nrf2, nuclear factor erythroid 2-related factor 2; OVA, ovalbumin; IL-5, pro-inflammatory cytokines interleukin 5; IL-13, pro-inflammatory cytokines interleukin 13; ERK, extracellular signal-related kinase; Keap1, Kelch-like ECH-associated protein 1; iNOS, inducible nitric oxide synthase; IL-1β, pro-inflammatory cytokines interleukin 1β; NO, nitric oxide; COX-2, cyclooxygenase-2; HSP70, 70-kDa heat shock protein; PARP, poly-ADP-ribose polymerase; JAK2, janus kinase 2; STAT3, signal transducer and activator of transcription 3; HSP90, 90-kDa heat shock protein.

## 6 Anti-infectious research trend

A growing body of research has highlighted *C. sappan* as a potent anti-infectious agent, effective against a wide range of microorganisms (Tables 4, 5). Seo et al. (2017) demonstrated the strong antitubercular properties of an ethyl acetate fraction derived from the heartwood of *C. sappan*. The active compound, 3-deoxysappanchalcone, exhibited significant activity against both drug-susceptible and drug-resistant strains of *Mycobacterium tuberculosis*. Additionally, it displayed partial synergy when combined with streptomycin and ethambutol, offering a promising avenue for tuberculosis treatment (Seo et al., 2017). In a study by Arjin et al. (2022), the bioactive compound brazilin from *C. sappan* was shown to inhibit viral infections, particularly in porcine reproductive and respiratory syndrome. Molecular docking predicted that brazilin had the highest binding energy with the receptor cysteine-rich domain 5 (SRCR5) of CD163. Brazilin effectively inhibited viral infection in MARC-145 cells, suggesting a potential application in antiviral therapies (Arjin et al., 2022). Puttipan et al. (2018) explored the antibacterial effects of the ethanolic extract and brazilin from *C. sappan* on *Streptococcus* mutans, a key pathogen in dental biofilm formation. Both the extract and brazilin demonstrated dose-dependent inhibition of biofilm formation. However, it was noted that brazilin exhibited higher cytotoxicity in fibroblasts, highlighting the need for careful dosage consideration in therapeutic applications (Puttipan et al., 2018). Nirmal and Panichayupakaranant (2014) investigated the antibacterial efficacy of brazilin and a brazilin-rich extract against anaerobic and aerobic bacteria. Their study revealed that Propionibacterium acnes was particularly susceptible to both treatments, with brazilin showing superior effectiveness, reinforcing its potential as an antimicrobial agent (Nirmal and Panichayupakaranant, 2014). Zuo et al. (2014) studied the synergistic effects of combining aminoglycoside antibiotics with *C. sappan* compounds, including brazilin, brazilein, and sappanone, against methicillin-resistant *Staphylococcus aureus* (MRSA). Among the compounds, brazilin displayed the strongest synergistic effect when used in combination with aminoglycosides, presenting a promising strategy to combat antibiotic resistance (Zuo et al., 2014). Jaisi et al. (2021) investigated the ethanolic extracts of twelve Thai medicinal herbs, including *C. sappan*, and their inhibitory effects on HIV-1 Viral Protein R (Vpr). Both the ethanolic extract and the isolated brazilin exhibited significant anti-Vpr activity, suggesting their potential role in HIV treatment strategies (Jaisi et al., 2021). In the realm of antimalarial research, Ma et al. (2015) reported the antimalarial and antiproliferative activities of compounds from *C. sappan* seeds. Two cassane diterpenes, Caesalsappanin G and H, were found to be highly effective against the chloroquine-resistant K1 strain of Plasmodium falciparum, with IC50 values of 0.78 and 0.52 μM, respectively. These compounds also demonstrated high selectivity indices, making them promising candidates for further development in antimalarial therapies (Ma et al., 2015). Zhu et al. (2017) identified two novel cassane diterpenoids, Caesalsappanin R and S, from *C. sappan* seeds. These compounds exhibited strong antiplasmodial activity, with Caesalsappanin R displaying the most potent effect, offering new possibilities for natural antimalarial agents (Zhu et al., 2017). Finally, Zuo et al. (2015) further explored the antibacterial properties of Protosappanins A and B from *C. sappan* heartwood. These compounds were found to be effective against MRSA and also demonstrated synergistic effects when combined with antibiotics such as amikacin and gentamicin, providing additional therapeutic options for combatting resistant bacterial infections (Zuo et al., 2015).

**TABLE 4:** Anti-infectious effect of *C. sappan* isolated compounds.

Compound/extract	Experimental model	Dose Duration	Efficacy	References
3-deoxysappanchalcone	*Mycobacterium tuberculosis* (H37Rv, XDR)	25 μg/mL	Antibacterial	Seo et al. (2017)
Brazilin	Molecular docking/PRRSV propagated in MARC-145 cells	2.5–10 μg/mL	Antiviral	Arjin et al. (2022)
Brazilin	*Streptococcus mutans*	125–500 μg/mL	Antibacterial	Puttipan et al. (2018)
Brazilin	*Propionibacterium acnes Staphylococcus aureus Staphylococcus epidermidis*	15.6, 31.3, 62.5 μg/mL 24, 72 h	Antibacterial	Nirmal and Panichayupakaranant (2014)
Brazilin Brazilein Sappanone B	methicillin-resistant *Staphylococcus aureus*	64–1,024 μg/mL 24 h	Antibacterial	Zuo et al. (2014)
Brazilin	TREx-HeLa-Vpr cells	10 μM	Antiviral	Jaisi et al. (2021)
Caesalsappanin G-H	*Plasmodium falciparum*	(IC_50_) 0.78 µM 0.52 µM	Antiplasmodial	Ma et al. (2015)
Caesalsappanin R	*Plasmodium falciparum*	(IC_50_) 3.6 µM	Antiplasmodial	Zhu et al. (2017)
Protosappanins A Protosappanins B	methicillin-resistant *Staphylococcus aureus*	24 h	Antibacterial	Zuo et al. (2015)

Abbreviation: ↓, downregulation; ↑, upregulation; Ø, inhibition; H37Rv and XDR, *Mycobacterium tuberculosis* strains; PRRSV, Porcine reproductive and respiratory syndrome virus; Vpr, Viral protein.

**TABLE 5:** Anti-infectious effect of *C. sappan* extracts.

Compound/extract	Experimental model	Dose Duration	Efficacy	References
Aqueous extract	*Staphylococcus aureus Staphylococcus epidermidis Propionibacterium acnes*	1 mg/mL 24 h	Antibacterial	Settharaksa et al. (2019)
Ethanolic extract	*Staphylococcus aureus, scherichia coli, Salmonella enteritidis, Vibrio parahaemolyticus*	200 μg/mL 24 h	Antibacterial	Pattananandecha et al. (2022)
Ethanolic extract	*Aeromonas hydrophila Favobacterium* sp. *Streptomyces* sp	10 mg/mL 24 h	Antibacterial	Techaoei (2022)
Ethanolic extract	*Elizabathkingia miricola*	0.0977–100 mg/mL 48 h	Antibacterial	Liu et al. (2023)
Ethanolic extract fraction	PRRSV propagated in MARC-145 cells	3.21–535.91 μg/mL 24 h	Antiviral	Arjin et al. (2021)
Ethyl acetate fraction	methicillin-resistant *Staphylococcus aureus*	10 mg/mL 24 h	Antibacterial	Jung et al. (2022)

Abbreviation: ↓, downregulation; ↑, upregulation; PRRSV, Porcine reproductive and respiratory syndrome virus.

Settharaksa et al. (2019) demonstrated the significant impact of time and temperature in obtaining an optimal aqueous extract of *C. sappan*, with all extracts displaying potent antibacterial activity. The extracts were particularly effective against *S. aureus*, *Staphylococcus* epidermidis, and Propionibacterium acnes, making them promising candidates for antimicrobial applications (Settharaksa et al., 2019). Pattananandecha et al. (2022) further investigated the efficacy of ethanolic extracts from *C. sappan* heartwood, identifying the 70% ethanol extract as the most potent. After 24 h of treatment at 200 μg/mL, this extract was able to completely inhibit 100% of all pathogens tested, highlighting its remarkable antimicrobial potential (Pattananandecha et al., 2022). In another study, Techaoei (2022) tested ten Thai medicinal plant extracts against three fish pathogenic bacteria, with *C. sappan* emerging as one of the most promising. It exhibited substantial antimicrobial activity in time-kill kinetics assays, showing potential as an effective treatment against fish pathogens (Techaoei, 2022). Liu et al. (2023) identified Elizabethkingia miricola as the causative agent of infectious diseases in farmed American bullfrogs and evaluated the antibacterial properties of 60 traditional Chinese herbal extracts. Among these, *C. sappan* showed the lowest minimal inhibitory concentration (less than 0.2 mg/mL), indicating its high efficacy. When used in multicomponent herbal mixtures, the results were further enhanced, showcasing its potential in combating bacterial infections (Liu et al., 2023). Arjin et al. (2021) assessed the antiviral activity of ethanolic fractions from *C. sappan* against porcine reproductive and respiratory syndrome virus (PRRSV). The most effective fraction contained a combination of the coumarin compound Byakangelicin and flavonoids such as Brazilin, Naringenin, and Brazilein. These compounds were identified as key bioactive constituents responsible for the antiviral effects, positioning *C. sappan* as a promising natural treatment for PRRSV (Arjin et al., 2021). Jung et al. (2022) conducted a comprehensive screening of 16 medicinal plants for their antibacterial properties, with *C. sappan* among the most effective. The ethyl acetate fraction of *C. sappan* heartwood demonstrated strong antibacterial activity, exhibiting a favorable balance between minimum inhibitory and bactericidal concentrations while maintaining low cytotoxicity, underscoring its potential for safe and effective antimicrobial applications (Jung et al., 2022).

## 7 Anti-cancer potential of *Caesalpinia sappan* research trend

Cancer remains a significant global health issue, (Kudamba A et al., 2023). with projections estimating that by 2050, the number of new cancer cases will reach 35 million annually (Bray et al., 2024; Kaur et al., 2024). Although various treatment methods—such as chemotherapy, immunotherapy, radiotherapy, and surgical resection—have been employed, the effectiveness of these treatments is often hampered by drug resistance and side effects (Fakudze NT et al., 2023). This has led to increasing interest in alternative therapies, particularly those derived from medicinal plants, which contain bioactive compounds with antitumor, antiproliferative, and cancer-preventive properties (Memarzia A et al., 2023). In recent years, isolated compounds and extracts from *C. sappan* have gained attention for their potential efficacy against a wide range of cancers (Tables 6–8). Kwak et al. (2021) investigated the mechanisms of action of 3-Deoxysappanchalcone, an isolated compound from *C. sappan*, in esophageal cancer cells. The compound induced apoptosis and G2/M cell cycle arrest through the JNK/p38 MAPK signaling pathway, showing potential as an effective anticancer agent (Kwak et al., 2021). Similarly, Zhao et al. (2019) found that 3-Deoxysappanchalcone inhibited colon cancer cell proliferation by targeting the T-lymphokine-activated killer cell-originated protein kinase (TOPK) pathway. This treatment also increased the expression of cell cycle proteins such as cyclin B1 and induced apoptosis via cleaved PARP, caspase-3, and caspase-7 (Zhao et al., 2019). In another study, Wudtiwai et al. (2023) demonstrated that Brazilein, a compound from *C. sappan*, significantly inhibited the viability of triple-negative breast cancer cells by inducing apoptosis. The compound suppressed epithelial-mesenchymal transition (EMT), programmed death-ligand 1 (PD-L1), and the AKT, NF-κB, and GSK3β/β-catenin signaling pathways (Wudtiwai et al., 2023). Handayani et al. (2022) explored the synergistic effect of combining brazilein with the chemotherapeutic drug doxorubicin in breast cancer cell lines. The combination enhanced cytotoxic activity and inhibited cell migration by downregulating HER2, p120, and Rac1, while suppressing MMP2 and MMP9 proteins (Handayani et al., 2022). In a similar study, Handayani et al. (2017) investigated the combination of brazilin and brazilein with cisplatin in colon cancer cells. The combination potentiated the cytotoxic effects of cisplatin, inducing apoptosis and causing cell cycle arrest in the S phase (Handayani et al., 2017). Jeon et al. (2014) found that brazilin disrupts IKK signaling, interfering with the formation of the proximal IL-1 receptor signaling complex, thereby offering another promising approach to cancer treatment (Jeon et al., 2014). Additionally, Kim et al. reported that brazilin inhibits the barrier-to-autointegration factor (BAF) in lung and neuroblastoma cell lines (Kim S. H. et al., 2015). The synergistic effects of brazilin with doxorubicin were further highlighted by Jenie et al. (2018), who demonstrated that this combination promotes apoptosis through G2/M phase cell cycle arrest and Bcl-2 suppression, enhancing the cytotoxicity of doxorubicin (Jenie et al., 2018). Meanwhile, Kang et al. (2018) revealed that brazilin induces autophagy via the forkhead box class O (FOXO)3A pathway and disrupts calcium homeostasis in osteosarcoma cells (Kang et al., 2018). In breast cancer cells, Chatterjee et al. showed that brazilin downregulates DNMT1 expression by recruiting p53 to the DNMT1 promoter, restoring p21 expression (Chatterjee et al., 2022). Suyatmi et al. (2022) demonstrated that brazilin induces intrinsic apoptosis in lung cancer cells by increasing p53, caspase-9, and caspase-3 expression (Suyatmi et al., 2022). Additionally, *in silico* tests conducted by Correia Soeiro et al. (2022) indicated that brazilin interacts with BAF1, a protein implicated in carcinogenesis (Correia Soeiro et al., 2022). Wang et al. (2021) isolated three compounds—Caesaterosides A, B, and C—from the seeds of *C. sappan*. These compounds exerted cytotoxic effects on colon, uterine, and liver cancer cell lines (Wang et al., 2021). Su et al. (2024) identified three more compounds—Caesalpanin D, E, and F—among which Caesalpanin D activated autophagy and reactive oxygen species (ROS) generation in pancreatic cancer cells (Su et al., 2024). Jin et al. (2022) isolated eight compounds from *C. sappan*, with compound 8 showing the most promising antitumor activity (Jin et al., 2022). Zhang et al. (2014) demonstrated that ethyl acetate extracts from *C. sappan* inhibited liver cancer cell mitosis, while isolated compounds such as sappanchalcone, brazilin, and butein exhibited cytotoxic activities against various cancer cells (Zhang et al., 2014). The extract also showed antitumor efficacy in a mouse model bearing S180 tumor cells. Tran et al. (2015) isolated five compounds from *C. sappan* seeds, with Phanginin D emerging as the most potent antitumor agent, inducing apoptosis through caspase-3 activation in leukemia cell lines (Tran et al., 2015). Bao et al. (2016) identified five cassane diterpenoids, Phanginins R‒T (1–3) and Caesalsappanins M and N (4 and 5), with compound 1 inducing apoptosis and cell cycle arrest in ovarian cancer cells (Bao et al., 2016). Sappanchalcone, isolated by Seo et al. (2020), was shown to induce apoptosis by activating caspases-3, -7, -8, -9, and PARP in colon cancer cells (Seo et al., 2020). Naik Bukke et al. (2018) confirmed the cytotoxic activity of *C. sappan* heartwood and leaf extracts against breast and lung cancer cells *in vitro*. An *in silico* study further suggested that one of the primary components of brazilin inhibits the BCL-2 protein, enhancing its anticancer potential (Naik Bukke et al., 2018). Furthermore, an *in silico* study confirmed that one of the primary components of the brazilin extracts inhibited the BCL-2 protein.Widodo et al. used crude extracts from the *C. sappan* stem to investigate its anticancer mechanisms in lung cancer cell lines. The treatment induced apoptosis and negatively regulated mitochondrial proteins associated with tumor cell survival (Widodo et al., 2022). In a separate study, Haryanti et al. (2022) evaluated the combination of *C. sappan* and Ficus septica extracts with doxorubicin, finding that the combination enhanced doxorubicin’s cytotoxic properties and inhibited cell migration in breast cancer cells (Haryanti et al., 2022). Ma et al. (2020) demonstrated that ethyl acetate extracts from *C. sappan* heartwood induce mitochondrial apoptosis by increasing cytochrome C expression and activating ROS in acute myeloid leukemia cells (Ma et al., 2020). Hung and Dat (2014) showed that methanolic extracts from the core of *C. sappan* exhibited the most promising cytotoxic results, inducing apoptosis in colon cancer cells via caspase-3 activation (Hung and Dat, 2014). The methanolic extract exhibited the most promising results, inhibiting proliferation and inducing apoptosis via activation of caspase-3 in colon cancer cell. Finally, two plant mixtures containing *C. sappan* extract—Hua-Zheng-Hui-Sheng-Dan and MANOSROI III—demonstrated potent antitumor activity. Hua-Zheng-Hui-Sheng-Dan reduced tumor volume in mice (Cao et al., 2015), while MANOSROI III induced apoptosis in colon cancer cells and (Manosroi A et al., 2015a) exhibited significantly higher inhibitory effects on cell proliferation than cisplatin and doxorubicin (Manosroi A et al., 2015b). Inprasitet al. (2014) also found that Sa-Tri-Lhung-Klod, another mixture containing *C. sappan* extract, exerted cytotoxic activity in ovarian and colon cancer cells (Inprasit et al., 2014). Li et al. (2021) demonstrated the antitumor efficacy of petroleum ether extracts from *C. sappan* roots and leaves, with the leaf and stem extract (SY②) showing the best results in reducing liver tumor size in mice and downregulating PCNA and VEGF expression (Li et al., 2021).

**TABLE 6:** Anti-cancer effect of *C. sappan* extracts.

Extract	Experimental model	Dose Duration	IC50	Efficacy	Mechanisms	References
Aqueous extract	MCF7	50–450 μg/mL 24 h	—	Cytotoxic effect	—	Naik Bukke et al. (2018)
	A549					
Aqueous extract	HL-60	5–100 μg/mL 48 h	>100 μg/mL	Cytotoxic effect	—	Hung and Dat (2014)
	HeLa		37.8 μg/mL			
	MCF-7		>100 μg/mL			
	LLC		>100 μg/mL			
	HepG2		78.6 μg/mL			
	KPL4		>100 μg/mL			
	HT-29		>100 μg/mL			
	KB		>100 μg/mL			
Chloroform extract	MCF7	50–450 μg/mL 24 h	—	Cytotoxic effect	—	Naik Bukke et al. (2018)
	A549					
Ethanolic extract	A549	10–320 μg/mL 24 h	45.19 μg/mL	Anti-cancer effect	↑ Bax, CDH15, FMOD, CLDN6, GUCY2C, SLC8A3, VARS2 ↓ Bcl-2, CD200R1, CEACAM7, RSPO4, ZBTB8B, ARMCX1, NXPH3, FABXW12, SOAT2	Widodo et al. (2022)
Ethanolic extract (w/doxorubicin too)	4T1	1.8, 10 μg/mL	9.3 μg/mL, 1.3 μg/mL	Anti-migrative effect	↓ p-IκBα, PARP-1, MMP-9	Haryanti et al. (2022)
Ethanolic extract	HL-60	5–100 μg/mL 48 h	68.5 μg/mL	Anti-cancer effect	—	Hung and Dat (2014)
	HeLa		39.2 μg/mL			
	MCF-7		>100 μg/mL			
	LLC		25.1 μg/mL			
	HepG2		>30 μg/mL			
	KPL4		>100 μg/mL			
	HT-29		>100 μg/mL			
	KB		>100 μg/mL			
Ethyl acetate extract	HL-60	0.025–3.2 mg/mL 48 h	0.19 mg/mL	Anti-cancer effect	↑ c-caspase-3, c-caspase-9	Ma et al. (2020)
Ethyl acetate extract	HGC-27	10 μg/mL		Anti-cancer effect		Zhang et al. (2014)
Hua-Zheng-Hui-Sheng-Dan mixing with *Caesalpinia sappan*	HeLa	Combination treatments		Anti-cancer effect	—	Cao et al. (2015)
Methanolic extract	HeLa	5–100 μg/mL 48 h	26.5 μg/mL	Anti-cancer effect	↑ c-caspase-3	Hung and Dat (2014)
Methanolic extract	MCF7	50–450 μg/mL 24 h	—	Cytotoxic effect	—	Naik Bukke et al. (2018)
	A549					
MANOSROI III mixing with *Caesalpinia sappan*	HT-29	Combination treatments		Anti-cancer effect	—	Manosroi A et al. (2015a)
MANOSROI III mixing with *Caesalpinia sappan*	Hep G2	Combination treatments		Cytotoxic effect	—	Manosroi A et al. (2015b)
Sa-Tri-Lhung-Klod mixing with *Caesalpinia sappan*	SKOV-3	Combination treatments		Cytotoxic effect	—	Inprasit et al. (2014)
	HeLa					

Abbreviations: ↓, downregulation; ↑, upregulation; Ø, interruption; c-, cleaved; p-phosphorylated; Bax, Bcl-2, associated X-protein; CDH15, cadherin 15 coding gene; FMOD, fibromodulin coding gene; CLDN6, claudin-6, coding gene; GUCY2C, guanylate cyclase 2C; SLC8A3, solute carrier family 8 member A3 coding gene; VARS2, valyl-tRNA, synthetase 2; Bcl-2, B-cell leukemia/lymphoma 2; CD200R1, CD200 Receptor 1 coding gene; CEACAM7, carcinoembryonic antigen-related cell adhesion molecule 7 coding gene; RSPO4, R-Spondin 4 coding gene; ZBTB8B, Zinc Finger And BTB, Domain Containing 8B coding gene; ARMCX1, Armadillo Repeat Containing X-Linked 1 coding gene; NXPH3, Neurexophilin 3 coding gene; FABXW12, F box protein-encoding gene; SOAT2 Sterol O-Acyltransferase 2 coding gene; IκBα, NF-kappa-B, inhibitor alpha; PARP-1, poly [ADP-ribose] polymerase-1; MMP-9, matrix metalloproteinase-9.

**TABLE 7:** Anti-cancer effect of *Caesalpinia sappan* extracts and isolated compounds using *in vivo* models.

Compound/Extract	Experimental model	Dose/Duration	Effects	References
Brazilin	S180 tumor cell-bearing mice model	400 mg/kg/8 days	↓ Tumor weight	Zhang et al. (2014)
Ethyl acetate extract	HL-60 cells-injected NOD/SCID mice	50, 100 mg/kg/40 days	↑ BMC ↓ Mice death, WBC, CD45, HPM, HE	Ma et al. (2020)
Sappanchalcone	S180 tumor cell-bearing mice model	200 mg/kg/8 days	↓ Tumor weight	Zhang et al. (2014)
Petroleum ether extract (roots)	H22 hepatoma-bearing mouse model	100, 325 mg/kg/12 days	↓ PCNA, VEGF	Li et al. (2021)
Petroleum ether extract (leaves/stems)		20, 65 mg/kg/12 days		

Abbreviations: ↓, downregulation; ↑, upregulation; NOD, Non-Obese Diabetic; SCID, severe combined immunodeficiency; WBC, white blood cells; CD45, pan-leukocyte marker; HPM, hepatosplenomegaly; HE, hepatic edema; BMC, bone marrow cellularity; PCNA, proliferating cell nuclear antigen; VEGF, vascular endothelial growth factor.

**TABLE 8:** Anti-cancer effect of *C. sappan* isolated compounds.

Compound/Extract	Experimental model	Dose Duration	IC50	Efficacy	Mechanisms	References
3-Deoxysappanchalcone	HCT-15	5–20 μM 24, 48, 72 h	NR	Anti-cancer effect	↑ c-PARP, c-caspase-3, c-caspase-7 ↓ ERKs, RSK, c-Jun	Zhao et al. (2019)
	HCT-116					
	SW620					
	DLD1					
3-Deoxysappanchalcone	KYSE 30	5–20 µM 48 h	19.8 µM	Anti-cancer effect	↑ JNK/p38 MAPK	Kwak et al. (2021)
	KYSE 410		12.2 µM			
Brazilein	MCF-7	0.31–5 mM 24, 48 h	23.74, 19.04 mM	Anti-cancer effect	↓ PD-L1, p-Akt, p-GSK3β, β-catenin, p-NF-κB	Wudtiwai et al. (2023)
	MDA-MB-231		58.96, 30.46 mM			
Brazilein	MCF-7/HER2	Either alone or combination with Doxorubicin		Anti-cancer effect	↓ HER2, Rac1, p120	Handayani et al. (2022)
Brazilein	WiDr	24 h	52 µM	Anti-cancer effect	—	Handayani et al. (2017)
Brazilin			41 µM			
Brazilin	HeLa	5–20 μM	NR	Anti-cancer effect	↓ IRAK1/4, TRAF6, MyD88, NF-κB	Jeon et al. (2014)
Brazilin	A549	3-30 µM 12 h	5 µM	Anti-cancer effect	↓BAF	Kim et al. (2015b)
Brazilin	MCF-7/HER2	Either alone, combination with Doxorubicin		Anti-cancer effect	↓ Bcl-2, HER2, p120, MMP-2, MMP-9	Jenie et al. (2018)
Brazilin	MG-63	5-20 µM 24 h	NR	Anti-cancer effect	↑ FOXO3A	Kang et al. (2018)
Brazilin	MCF-7	0.1–50 µM 24, 48 h	NR	Anti-cancer effect	↑ p38MAPK	Chatterjee et al. (2022)
Brazilin	A549	5–100 μg/mL 24 h	43 μg/mL 24 h	Anti-cancer effect	↑ p53, caspase-9, caspase-3	Suyatmi et al. (2022)
Brazilin (analogs)	Molecular docking	—		Anti-cancer effect	↓BAF1	Correia Soeiro et al. (2022)
Brazilin	HepG2	24 h	11.91 μg/mL	Anti-cancer effect	↓TNFα/NF-κB	Zhang et al. (2014)
	H522		3.7 μg/mL			
	COLO 205		6.47 μg/mL			
Butein	HepG2		1.78 μg/mL		↓IL-6/STAT3	
	H522		10.40 μg/mL			
	COLO 205		3.95 μg/mL			
Caesateroside A	HepG-2	5–100 µM 24, 48 h	45.3, 34.6 µM	Cytotoxic effect	—	Wang et al. (2021)
	HeLa		44.2, 32.5 µM			
	L-02		>100 µM			
Caesateroside B	HepG-2		35.7, 18.3 µM			
	HeLa		18.2, 12.2 µM			
	L-02		>100, 86.9 µM			
Caesateroside C	HepG-2		>100, 72.7 µM			
	HeLa		>100, 67.8 µM			
	L-02		>100 µM			
Cassane compound 1	L02	1–50 μM 48 h	>50 μM	Cytotoxic effect	—	Jin et al. (2022)
	HepG2		13.48 μM			
	MCF-7		27.37 μM			
	A549		25.37 μM			
	Caco-2		35.13 μM			
Cassane compound 2	L02		>50 μM			
	HepG2		18.91 μM			
	MCF-7		29.67 μM			
	A549		42.20 μM			
	Caco-2		32.33 μM			
Cassane compound 4	L02		>50 μM			
	HepG2		44.88 μM			
	MCF-7		27.53 μM			
	A549		36.37 μM			
	Caco-2		43.33 μM			
Cassane compound 5	L02		>50 μM			
	HepG2		>50 μM			
	MCF-7		35.53 μM			
	A549		41.50 μM			
	Caco-2		>50 μM			
Cassane compound 6	L02		>50 μM			
	HepG2		>50 μM			
	MCF-7		43.37 μM			
	A549		23.40 μM			
	Caco-2		31.71 μM			
Cassane compound 8	HepG2		7.82 μM	Cytotoxic effect	Ø G0/G1 phase	
Cassane compound 10	L02		>50 μM	Cytotoxic effect	—	
	HepG2		27.25 μM			
	MCF-7		>50 μM			
	A549		46.87 μM			
	Caco-2		29.95 μM			
Cassane compound 11	L02		>50 μM			
	HepG2		29.78 μM			
	MCF-7		>50 μM			
	A549		37.29 μM			
	Caco-2		>50 μM			
Compound 4	PANC-1	1.25–10 µM 48 h	5 and 10 µM	Anti-cancer effect	↓AMPK/mTORC1	Su et al. (2024)
Phanginin I	HL-60	1-100 μM 24 h	16.4 μM	Cytotoxic effect	—	Tran et al. (2015)
	HeLa		28.1 μM			
	MCF-7		>100 μM			
	LLC		>100 μM			
Phanginin A	HL-60		19.2 μM			
	HeLa		37.2 μM			
	MCF-7		>100 μM			
	LLC		>100 μM			
Phanginin D	HL-60		11.7 μM	Anti-cancer effect	↑ c-caspase-3	
Phanginin H	HL-60		22.5 μM	Cytotoxic effect		
	HeLa		>50 μM			
	MCF-7		>100 μM			
	LLC		42.5 μM			
Phanginin J	HL-60		46.9 μM			
	HeLa		>50 μM			
	MCF-7		>100 μM			
	LLC		>100 μM			
Phanginins R	A549	5, 10, 20 μM 24 h	NR	Anti-cancer effect	↑ p53, c-PARP ↓Bcl-2	Bao et al. (2016)
Sappanchalcone	HepG2	24 h	0.91 μg/mL	Cytotoxic effect	Ø G2/M phase	Zhang et al. (2014)
	H522		1.31 μg/mL			
	COLO 205		21.76 μg/mL			
Sappanchalcone	HCT116	10–50 μM 48 h	37.33 μM	Anti-cancer effect	↑ c-caspase-3, c-caspase-7, c- caspase-8, c-caspase-9, c-PARP	Seo et al. (2020)
	SW480		54.23 μM			

Abbreviations: ↓, downregulation; ↑, upregulation; Ø, interruption/inhibition; NR, not reported; c-, cleaved; p-, phosphorylated; PARP, poly-ADP-ribose polymerase; ERKs, extracellular signal-regulated kinase; RSK, ribosomal S6 kinase; c-Jun, Jun protein; JNK/p38 MAPK, Jun N-terminal kinases and p38 mitogen-activated protein kinases; PD-L1, programmed death-1; Akt, protein kinase B; GSK3β, glycogen synthase kinase 3β; NF-κB, nuclear factor kappa-light-chain-enhancer of activated B cells; HER2, human epidermal growth factor receptor 2; RAC1, ras-related C3 botulinum toxin substrate 1; p120, catenin delta-1; IRAK1/4, interleukin-1, receptor-associated kinase 4; TRAF6, TNF, receptor-associated factor 6; MyD88, myeloid differentiation primary response 88; BAF, barrier-to-autointegration factor; Bcl-2, B-cell lymphoma 2; MMP-2, matrix metalloproteinase-2; MMP-9, matrix metalloproteinase-9; FOXO3A, forkhead box transcription factor O 3a; p38MAPK, p38 mitogen-activated protein kinase; p53, tumor protein p53; TNFα, tumor necrosis factor alpha; IL-6, pro-inflammatory cytokines interleukin 6; STAT3, signal transducer and activator of transcription 3; AMPK, AMP-activated protein kinase; mTORC1, mechanistic target of rapamycin complex 1.

## 8 Anti-cancer of *C. sappan* crude extract and the isolated compounds research trend

**FIGURE 4:**
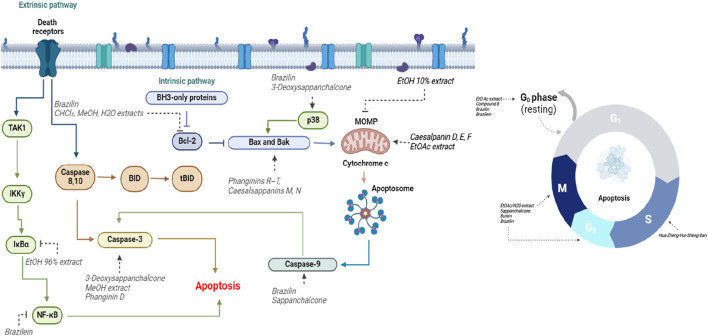
Anti-Cancer Effect of *C. sappan* Extracts and Isolated Compounds. Apoptosis occurs through two main pathways: the extrinsic and intrinsic pathways. The extrinsic pathway involves the activation of death receptors, leading to the recruitment of caspases-8 and -10, which then activate caspase-3. Brazilin and various *C. sappan* extracts have been shown to activate this pathway, triggering caspase-3, a key enzyme responsible for apoptosis. Compounds like 3-Deoxysappanchalcone, the methanol MeOH extract and Phanginin D increased caspases-3 in tumor cells. The intrinsic pathway is regulated by the Bcl-2 family of proteins such as Bax and Bak, which cause the release of cytochrome c from mitochondria, leading to apoptosis. Compounds such as Brazilin, 3-Deoxysappanchalcone, Phanginins R-T, Caesalsappanins M, N, Caesalpanin D, E, F, EtOAc extract and Sappanchalcone demonstrated this effect. Additionally, compounds such as EtO Ac, Compound B, Brazilin, Brazilein, Sappanchalcone, Butein, and Hua-Zhen-Hui-Sheng-Dan disrupted the cell cycle at different phases. Abbreviations:↓, upregulation; , T inhibit; CHCl₃, chloroform; MeOH, methanol; H_2_O, water; EtOAc, ethyl acetate; TAK1, Transforming growth factor-β activated kinase 1; IκBα, inhibitors of NF-κB; NF-κB, Nuclear factor kappa-light-chain-enhancer of activated B cells; Bcl-2, B-cell lymphoma-2; Bax, Bcl-2–associated X protein; BH3, 3 Bcl-2 homology regions; MOMP, mitochondrial outer membrane permeabilization; BID, BH3-interacting domain death agonist; tBID, truncated p15 BID.

Cancer therapies often focus on modulating cell death pathways, such as apoptosis, necrosis/necroptosis, and autophagy, which are crucial for the development of novel chemotherapeutic drugs (Hadian and Stockwell, 2023). Among these, apoptosis—commonly referred to as programmed cell death or “cell suicide”—is particularly significant (Merve Kulbay et al., 2021). Two major pathways mediate apoptosis: the intrinsic pathway, induced by cellular stress or regulated by B-cell lymphoma-2 (Bcl-2) family proteins, and the extrinsic pathway, initiated by death receptor ligands (Zhu M et al., 2023). Notably, *C. sappan* has demonstrated potent antitumor activity by activating the apoptosis pathway (Figure 4). Research shows that fractions of the crude extract and isolated compounds such as Phanginins R-T and Caesalsappanins M and N from C. sappan have the ability to increase the expression of Bcl-2 family proteins, which regulate mitochondrial outer membrane permeabilization, a key step in apoptosis. Apoptosis is facilitated by intracellular proteases called caspases, which are critical for initiating and executing the apoptotic process. These caspases are divided into initiator caspases (caspase 8, 9, and 10) and effector caspases (caspase 3, 6, and 7) (Kesavardhana and Kanneganti, 2020). Initiator caspases amplify apoptotic signals, subsequently activating effector caspases, which cleave cellular proteins to drive apoptosis (Kesavardhana and Kanneganti, 2020). Activation of caspase-9 indicates the intrinsic apoptosis pathway, while caspase-8 activation signifies the extrinsic pathway (Bock and Tait, 2020). Isolated compounds from *C. sappan*, including 3-Deoxysappanchalcone, Brazilin, and Sappanchalcone, have been shown to increase caspase-3 expression, with Sappanchalcone and Brazilin also promoting caspase-9 activation, underscoring their role in intrinsic apoptosis. Mitochondria play a central role in this process. Bcl-2 family proteins regulate mitochondrial outer membrane permeabilization, which triggers apoptosis via the release of cytochrome c (Chu et al., 2021). This mitochondrial mechanism was observed in the ethanolic extract of *C. sappan*, further emphasizing the plant’s pro-apoptotic properties. Additionally, autophagy is another important cell death pathway, although its relationship with cancer is complex. While autophagy can act as a survival mechanism for cancer cells, evidence also suggests it can suppress tumor growth under certain conditions (Li X and Ma, 2020). One crucial factor in autophagy is the phosphorylation of FOXO3, which induces autophagy (Ashrafizadeh et al., 2022). This mechanism has been demonstrated by the compounds Brasilin, Caesalpanin D, E, and F, isolated from *C. sappan*.

Furthermore, the isolated compounds and fractions from *C. sappan* have been shown to induce cell cycle arrest in tumor cells, a vital mechanism in controlling tumor progression. While mitosis is necessary for normal cell division, it becomes dysregulated in cancer cells, leading to uncontrolled proliferation (Huang et al., 2022). *Caesalpinia sappan* compounds target key regulatory proteins involved in mitosis, thereby inducing cell cycle arrest and promoting tumor cell death (Paier CRK et al., 2018).

## 9 Anti-diabetic effect of *C. sappan* extracts and isolated compounds research trend

**FIGURE 5:**
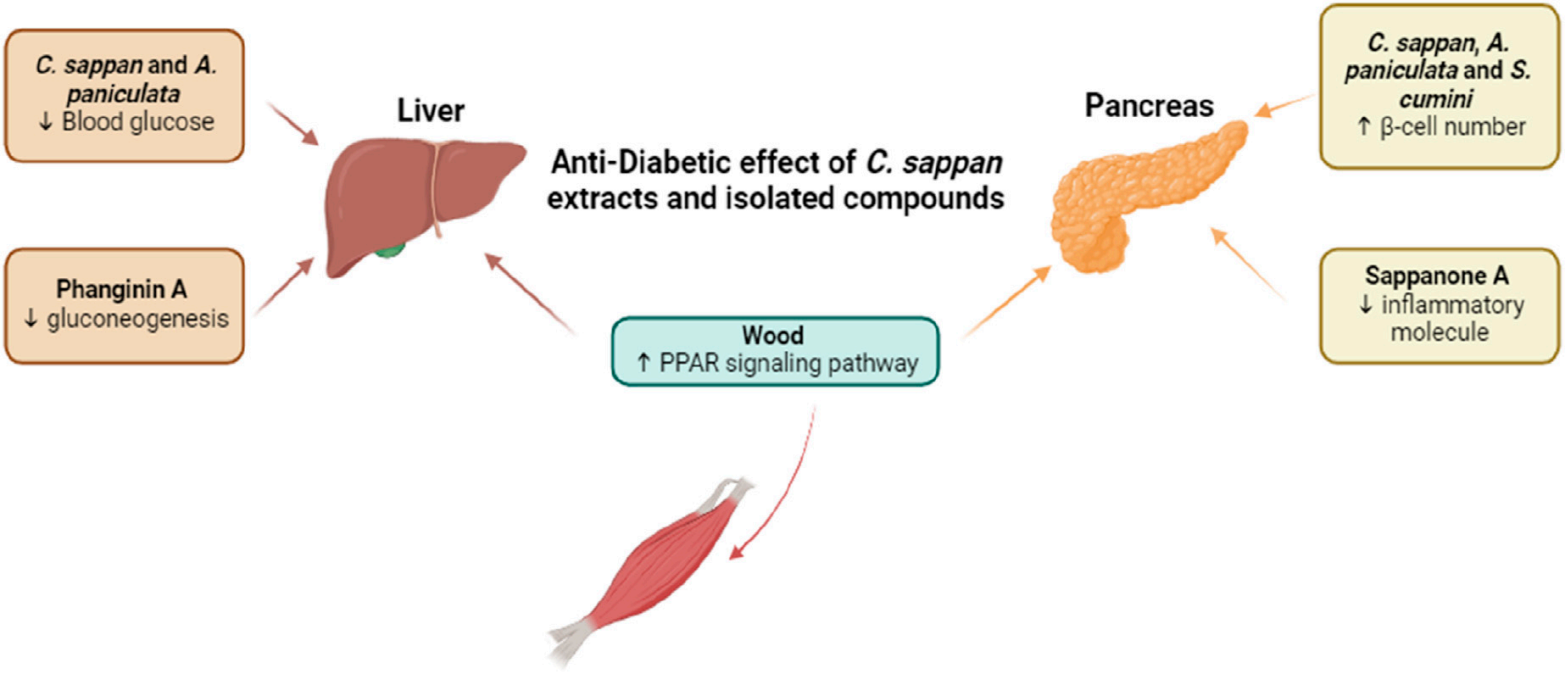
Anti-Diabetic Effect of *C. sappan* Extracts and Isolated Compounds. *C sappan* extracts and compounds target PPARs, nuclear receptor proteins that function as transcription factors and are key targets for new diabetes treatments. *Caesalpinia sappan* wood extract has been shown to act on this pathway. Isolated compounds such as Phanginin A and Sappanone A, along with combined treatments involving *C. sappan*, demonstrate mechanisms that lower blood glucose, reduce gluconeogenesis and inflammation, and increase β-cell activity in diabetic models. Abbreviations: ↓, upregulation; ↑, downregulation; PPAR, Peroxisome proliferator-activated receptors.

Diabetes mellitus, a group of metabolic disorders characterized by persistent hyperglycemia, remains a significant global health challenge (Harreiter and Roden, 2023). Conventional treatments, such as oral hypoglycemic agents and insulin injections, offer temporary control of blood glucose levels but fall short in preventing long-term complications and often come with adverse side effects (Sun et al., 2021). This has led to growing interest in exploring alternative therapies, particularly those derived from medicinal plants, which offer benefits like enhanced safety, specific modes of action, and improved metabolic regulation. Research on compounds and extracts from *C. sappan* has shown potential in addressing diabetic symptoms and complications (Figure 5; Table 9). Studies conducted by Wediasari et al. (2020) demonstrated the potential of a combination therapy involving *C. sappan* and Andrographis paniculata, which yielded promising *in vivo* results. The combination significantly lowered blood glucose levels and increased pancreatic β-cell regeneration in diabetic rats, indicating its therapeutic potential for managing diabetes (Wediasari et al., 2020). Masaenah et al. (2021) further explored the combined effects of Andrographis paniculata, Syzygium cumini, and *C. sappan* extracts in diabetic rats. This treatment not only reduced fasting blood glucose levels but also moderately improved pancreatic β-cell function and maintained normal lipid profiles, without causing toxicity at the administered doses (Masaenah et al., 2021). These findings underscore the safety and efficacy of *C. sappan* as part of combination therapies for diabetes management. Wu et al. (2022) focused on Phanginin A, a compound isolated from *C. sappan* seeds, which demonstrated the ability to inhibit hepatic gluconeogenesis by increasing SIK1 phosphorylation. This mechanism significantly improved hyperglycemia in type 2 diabetic mice, suggesting that Phanginin A could be a valuable candidate for diabetes treatment (Wu et al., 2022). Wang et al. (2022) investigated the effects of Sappanone A, a compound derived from *C. sappan* heartwood, and found that it prevented diabetes-related complications such as renal inflammation and fibrosis. These effects were attributed to its inhibition of the NF-κB pathway, both *in vitro* and *in vivo*, highlighting its potential to alleviate diabetic complications at the molecular level (Wang et al., 2022). In an *in silico* study, Adnan et al. (2022) identified several bioactive compounds present in the crude extract of *C. sappan* wood, particularly Fisetin tetramethyl ether, which was found to activate the peroxisome proliferator-activated receptor (PPAR) signaling pathway. This pathway plays a crucial role in glucose homeostasis, further supporting the anti-diabetic potential of *C. sappan* (Adnan et al., 2022). The growing body of research on *C. sappan* extracts and isolated compounds provides compelling evidence of their potential as effective and safe alternatives for diabetes treatment. These findings pave the way for further studies to explore *C. sappan*’s therapeutic applications, particularly in combination with other medicinal plants.

**TABLE 9:** Anti-Diabetic effect of *C. sappan* extracts and isolated compounds.

Compound/Extract	Experimental model	Dose Duration	Efficacy	Mechanisms	References
Fisetin tetramethyl ether	Molecular Docking Assay		Strong binding affinity	PPAR pathway	Adnan et al. (2022)
Mixing with *C. sappan* and *Andrographis paniculata*	Sprague Dawley rats	100, 200 mg/kg 7 days	Antihyperglycemic effect	↑ β-cells ↓ BG	Wediasari et al. (2020)
Mixing with *C. sappan*, *Andrographis paniculata* and *Syzygium cumini*	Sprague Dawley rats	75, 150 mg/kg 7 days	Antihyperglycemic effect	↑ β-cells ↓ FBG, LDL, cholesterol	Masaenah et al. (2021)
Phanginin A derivative	C57BL/6J mice	2.5, 5, 10 μM	Anti-hepatic gluconeogenesis	↑ p-SIK1	Wu et al. (2022)
Sappanone A	C57BL/6J mice	10, 20, 30 mg/kg 2x per day	Inhibition of kidney inflammation and fibrosis	↑ IκBα ↓ TGF-β1, Col-IV, IL-1β, TNF-α, NF-κB	Wang et al. (2022)

Abbreviations: ↓, upregulation; ↑, downregulation; BG, blood glucose; FBG, fasting blood glucose; LDL, low-density lipoproteins, p-SIK1, phosphorylated salt-induced kinase 1; IκBα, nuclear factor of kappa light polypeptide gene enhancer in B-cells inhibitor alpha; TGF-β1, transforming growth factor beta-1; Col-IV, Collagen IV; IL-1β, interleukin-1, beta; TNF-α; tumor necrosis factor-alpha; NF-κB, nuclear factor kappa-light-chain-enhancer of activated B cells; PPAR, peroxisome proliferator-activated receptor.

## 10 The effect of *C. sappan* extracts on cardiovascular disease research trend

**FIGURE 6:**
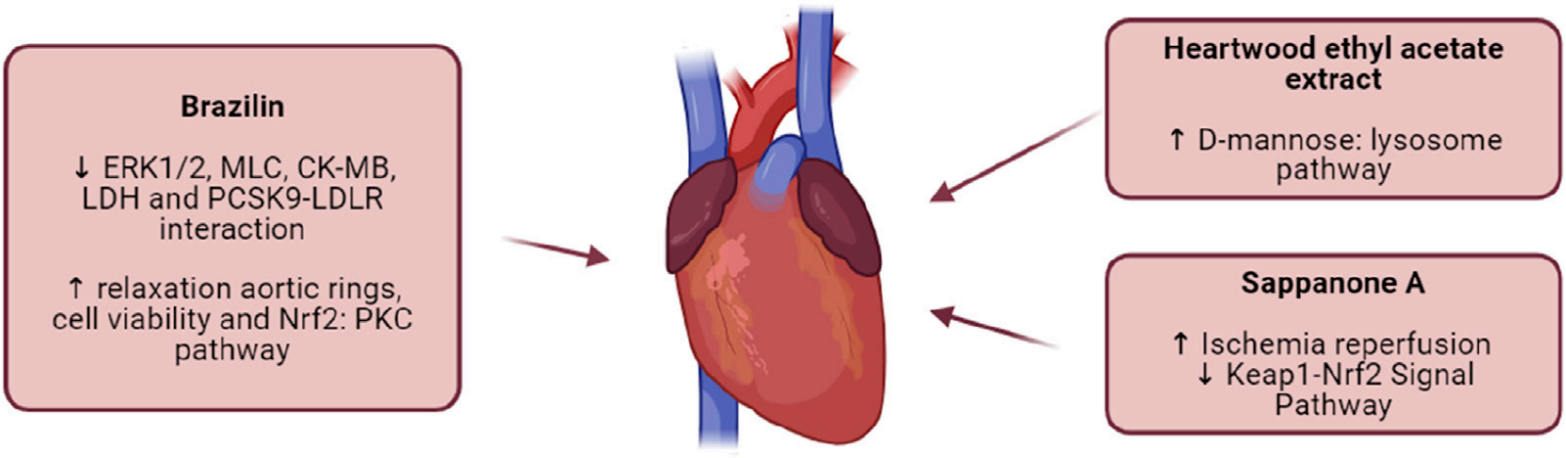
Cardiovascular Effect of *C. sappan* Extracts and Isolated Compounds. Myocardial injury and inflammation often lead to oxidative stress. The Nrf2 pathway, which regulates the PKC pathway and has antioxidant functions, is activated by Brazilin to limit oxidative stress. Sappanone A has shown efficacy in treating ischemia-reperfusion injury, a key factor in cardiovascular disease. The ethyl acetate extract of *C. sappan* heartwood has demonstrated improvement in ischemia and heart failure through the lysosomal pathway via autophagy. Abbreviations: ↓, upregulation; ↑, downregulation; ERK1/2, extracellular signal-regulated kinase 1/2; MLC, mixed lymphocyte culture; CK-MB, Creatine Kinase-MB; LDH, Lactate Dehydrogenase; Nrf2, nuclear factor erythroid 2-related factor 2; PKC, protein kinase C; KEAP1, kelch-like ECH-associated protein 1; PCSK9, Proprotein convertase subtilisin/kexin type-9; LDLR, LDL receptor.

Cardiovascular diseases, which encompass conditions such as heart attacks and strokes, primarily result from pathological changes in the heart or blood vessels (Nitsa A et al., 2018). These diseases are the leading cause of mortality worldwide, with atherosclerotic cardiovascular disease being the most prevalent contributor to overall deaths (Goldsborough E and Blaha, 2022). Despite significant advancements in treatment, cardiovascular diseases continue to pose a major global public health challenge, resulting in substantial social and economic burdens (Gao and Hou, 2023). Recent studies suggest that *C. sappan* extracts and its compounds could offer cardioprotective benefits, making it a promising avenue for cardiovascular health management (Figure 6; Table 10). Yan et al. (2015) demonstrated that Brazilin, a bioactive compound isolated from *C. sappan*, induces relaxation of aortic rings in rats, suggesting its potential to improve vascular function (Yan et al., 2015). This vasodilatory effect highlights Brazilin’s ability to enhance cardiovascular health by promoting healthy blood flow and reducing the strain on the cardiovascular system. Further research by Qi et al. (2021) revealed that Brazilin plays a protective role in preventing myocardial ischemia-reperfusion injury in rats. This cardioprotective effect is attributed to its activation of the Nrf2 pathway via protein kinase C (PKC), which underscores Brazilin’s potential to mitigate heart damage during ischemic events (Qi et al., 2021). Iqbal et al. (2023) explored Brazilin’s impact on proprotein convertase subtilisin/kexin type 9 (PCSK9), a key regulator of cholesterol metabolism. Elevated levels of PCSK9 are linked to an increased risk of cardiovascular disease. The study demonstrated that Brazilin effectively regulates PCSK9 levels, making it a promising candidate for cardiovascular disease prevention by helping to manage cholesterol levels and reduce atherosclerosis risk (Iqbal et al., 2023). Liu et al. (2022) investigated the effect of an ethyl acetate extract from *C. sappan* heartwood in an atherosclerosis model. The extract enhanced D-mannose production through the lysosomal pathway and improved lysosomal function in mice. This finding suggests that *C. sappan* may aid in combating atherosclerosis by promoting healthier cellular function and reducing plaque buildup (Liu et al., 2022). Lastly, Shi et al. (2020) focused on Sappanone A, another compound isolated from *C. sappan*, and its potential to prevent myocardial ischemia-reperfusion injury. The study found that Sappanone A modulates the Nrf2 pathway via PKC and PI3K, providing therapeutic benefits against myocardial injuries caused by ischemia (Shi et al., 2020). These studies highlight the potential of *C. sappan* and its compounds as promising natural interventions for cardiovascular disease. As a natural resource with diverse bioactive compounds, *C. sappan* may offer novel approaches for the prevention and treatment of cardiovascular conditions, contributing to improved heart health and overall well-being.

**TABLE 10:** Cardiovascular effect of *C. sappan* extracts and isolated compounds.

Compound/Extract	Experimental model	Dose/Duration	Mechanisms	References
Brazilin	Specific pathogen-free Sprague-Dawley male rats	83.51 μmol/L	↑ relaxation in rat aortic rings ↓ p-ERK1/2, MLC	Yan et al. (2015)
Brazilin	H9c2 cells	25 µM	↑ cell viability ↓ CK-MB, LDH	Qi et al. (2021)
	male Wistar rats	25 mg/kg	↑ Nrf2: PKC pathway	
Brazilin	PCSK9-LDLR Binding Assay	2.19 μM	↓ PCSK9-LDLR interaction	Iqbal et al. (2023)
Ethyl acetate extract	ApoE−/− mice	3.125 g kg^−1^ d^−1^	↑ D-mannose: lysosome pathway	Liu et al. (2022)
Sappanone A	male Wistar rats	20 mg/kg	↑ Keap1-Nrf2 Signal Pathway ↓ Ischemia Reperfusion	Shi et al. (2020)

Abbreviations: ↓, downregulation; ↑, upregulation; ERK1/2, extracellular signal-regulated kinase 1/2; MLC, mixed lymphocyte culture; CK-MB, Creatine Kinase-MB; LDH, lactate dehydrogenase; Nrf2, nuclear factor erythroid 2-related factor 2; PKC, protein kinase C; KEAP1, kelch-like ECH-associated protein 1; PCSK9, Proprotein convertase subtilisin/kexin type-9; LDLR, LDL, receptor.

## 11 Therapeutic effect of *C. sappan* extract and isolated compounds on joint-related diseases research trend

Several studies have highlighted the potential of *C. sappan* for the treatment of joint-related diseases, further showcasing the plant’s extensive range of health benefits (Table 11). Jung et al. (2015a) demonstrated that Brazilin, an active compound isolated from *C. sappan*, significantly reduced the arthritis index score and alleviated acute inflammatory paw edema in type-II collagen-induced arthritis (CIA) mice (Jung et al., 2015a). Notably, Brazilin prevented joint destruction and surface erosion, improved bone health, and significantly reduced the serum levels of key inflammatory cytokines such as TNF-α, IL-1β, and IL-6, thereby attenuating CIA in the model. Similarly, Jung et al. (2015b) also reported the effects of sappanchalcone, another compound from *C. sappan*, which was shown to decrease clinical arthritis severity and inflammatory paw edema in CIA mice. Treatment with sappanchalcone preserved bone mineral density and trabecular structure while significantly lowering the levels of pro-inflammatory cytokines, including TNF-α, IL-6, and IL-1β. Weinmann et al. (2018) found that Brazilin reduced glycosaminoglycan (GAG) loss in cartilage explants stimulated with IL-1β and TNF-α, protecting cartilage from degradation. Analysis of the NF-κB pathway in chondrocytes revealed that NFKB1/p50 plays a key role in regulating Brazilin’s anti-inflammatory actions. Brazilin was able to suppress the IL-1β-induced upregulation of osteoarthritic (OA) markers and the activation of NFKB1/p50 in chondrocytes, suggesting a protective effect against OA progression. Kim et al. further explored the impact of Brazilin on bone health, showing that it inhibited RANKL-mediated osteoclast differentiation in RAW264.7 cells without causing cytotoxicity (Kim J. et al., 2015). Brazilin downregulated the expression of key osteoclast markers, including tartrate-resistant acid phosphatase (TRAP), NFATc1, matrix metalloproteinase-9 (MMP-9), and cathepsin K, while also reducing RANKL-induced expression of pro-inflammatory and osteoclastogenic factors such as iNOS, COX-2, TNF-α, and NF-κB p65. Additionally, in a lipopolysaccharide (LPS)-induced osteoporosis model, Brazilin was found to attenuate bone loss *in vivo*, further highlighting its bone-protective potential. Choo et al. (2017) focused on the role of Sappanone A, another compound from *C. sappan*, in preventing inflammation-induced bone loss. Sappanone A inhibited RANKL-induced osteoclastogenesis and bone resorption by targeting the AKT/glycogen synthase kinase-3β (GSK-3β) signaling pathway and NFATc1, suppressing downstream target genes such as CtsK, TRAP, MMP-9, DC-STAMP, and OSCAR. This study reinforced the compound’s potential as a therapeutic agent in preventing osteoclast-mediated bone diseases. Finally, Lee S. et al. (2015) reported that Brazilin enhanced autophagic flux in rheumatoid arthritis fibroblast-like synoviocytes (RA FLS), as evidenced by increased autophagosome formation and elevated levels of lipidated LC3 (LC3-II), primarily mediated by increased ROS production (Lee S. et al., 2015). Additionally, Brazilin suppressed NF-κB activation and attenuated the inflammatory response under autophagy-inducing conditions in RA FLS, indicating its potential in managing rheumatoid arthritis by modulating autophagy and inflammation. These studies collectively highlight the multifaceted potential of *C. sappan* extracts and isolated compounds in treating joint-related diseases by modulating inflammatory and bone-degrading pathways, reinforcing its promise as a natural therapeutic agent for joint health.

**TABLE 11:** Effect of *C. sappan* extracts and isolated compounds against jointed-related diseases.

Compound/Extract	Experimental model	Dose/Duration	Mechanisms	References
Brazilin	RA FLS, HDF , NIH3T3, MEF, COS-7 cells	5, 10, 25 μg/mL: RA FLS (3,6,12, 24,48 h)	↑LC3-II	Lee et al. (2015b)
		25 μg/mL (3,6,12, 24 h)	↑ROS	
		(3, 6,12, 24,48 h)	↓NF-κB	
Brazilin	DBA/1J mice	10 mg/kg/mice	↓TNF-α, IL-1β, IL-6	Jung et al. (2022)
Brazilin	osteochondral explants	10 μg/mL (7 days)	↓GAG	Weinmann et al. (2018)
	PCs, C-28/I2 cells, SW1353 cells	10 μg/mL (1 h)	↓MMP-1, MMP-3, MMP-13, NFKB1/p50	
Brazilin	RAW264.7 cells	1, 5, 10 μg/mL	↓ TRAP, NFATc1, MMP-9, CtsK, iNOS, COX-2, TNF-α, IL-6, p-ERK, NF-Κb, p-65	Kim et al. (2015a)
	ICR mice	100 mg/kg/mice	↓bone loss	
Sappanone A	BMMs	3, 10, 30 μM (7 days) 3, 10, 30 μM (30 min) 30 μM (24, 48 h)	↓osteoclasts, osteoclast actin-ring, NFATc1, AKT/GSK-3β, CtsK, TRAP, DC-STAMP, MMP-9, OSCAR p-AKT NFATc1	Choo et al. (2017)
	Recombinant mouse M- CSF treatment in ICR mice	50 mg/kg/mice 9 days	↓Bone loss, osteoclasts	
Sappanchalcone	DBA/1J mice	10 mg/kg/mice	↓TNF-α, IL-6, IL-1β	Jung et al. (2015b)

Abbreviations: ↓, downregulation; ↑,upregulation; HDF, human dermal fibroblast, MEF, mouse embryonic fibroblast; TNF-α, tumor necrosis factor alpha; 1L-1β, pro-inflammatory cytokines interleukin-1, beta; IL-6, pro-inflammatory cytokines interleukin 6; GAG, glycosaminoglycan; PCs, primary chondrocytes; MMP-1, matrix metalloproteinase 1; MMP-3, matrix metalloproteinase 3; MMP-13, matrix metalloproteinase 13; ROS, reactive oxygen species; TRAP, tartrate-resistant acid phosphatase; NFATc1, nuclear factor of activated T-cells, cytoplasmic 1; MMP-9, matrix metalloproteinase 9; iNOS, inducible nitric oxide synthase; COX-2, cyclooxygenase-2; ERK, extracellular signal-regulated kinases; NF-κB, nuclear factor kappa-light-chain-enhancer of activated B cells; BMMs, bone marrow macrophages; AKT/GSK-3β, CtsK, cathepsin K; AKT/glycogen synthase kinase-3β; LPS, lipopolysaccharide; RA FLS, rheumatoid arthritis fibroblast-like synoviocytes; ROS, reactive oxygen species.

## 12 Discussion

### 12.1 Anti-cancer properties of *C. sappan*

The therapeutic potential of *C. sappan* has garnered significant attention for its anti-cancer properties across various pharmacological areas. Compounds such as 3-Deoxysappanchalcone, Brazilein, Caesaterosides A, B, and C, Cassane compounds, Sappanchalcone, and Butein have demonstrated strong anti-cancer activity by inducing apoptosis, arresting the cell cycle, or inhibiting metastasis. Additionally, extraction solutions like ethanolic, ethyl acetate, and methanolic extracts have exhibited potent anti-cancer effects in both *in vitro* and *in vivo* models. While prior research has largely focused on individual compounds, our review emphasizes the collective effects of these compounds and extracts. We demonstrate how these compounds, acting together, can target multiple cancer pathways, offering a more comprehensive approach to cancer therapy.

### 12.2 The growing importance combination therapy with herbal medicine

The integration of herbal medicine in combination therapies for treating tumors is gaining increasing significance. Numerous studies have demonstrated that combining herbal medicine with conventional chemotherapy can enhance efficacy, improve survival rates, and mitigate the side effects of standard cancer treatments (Efferth et al., 2008; Lam et al., 2010; Man et al., 2015). Traditional herbal formulations like Hua-Zheng-Hui-Sheng-Dan, MANOSROI III, and Sa-Tri-Lhung-Klod, all of which include *C. sappan*, have shown remarkable therapeutic results. These formulations are deeply rooted in ethnopharmacology and have been traditionally used in various cultures for their healing properties. For instance, Hua-Zheng-Hui-Sheng-Dan, a classic formulation in traditional Chinese medicine, is used to improve blood circulation and reduce tumor burden. Similarly, MANOSROI III, developed in Thailand, integrates *C. sappan* with other native herbs to enhance its anti-inflammatory and anticancer effects (Manosroi A et al., 2015b), reflecting its long-standing use in Thai traditional medicine. Sa-Tri-Lhung-Klod, another Thai formulation, has been historically utilized to manage inflammation and promote overall wellbeing, aligning with its observed anticancer properties (Inprasit et al., 2014). These formulations act synergistically, targeting multiple cancer pathways and addressing the limitations of single-agent therapies. For example, Hua-Zheng-Hui-Sheng-Dan and Sa-Tri-Lhung-Klod, when combined with *C. sappan*, have been reported to significantly suppress tumor growth and improve patient outcomes (Li et al., 2021). This highlights their dual ethnopharmacological and therapeutic value. Our review contributes by exploring the synergistic potential of *C. sappan* in combination with these traditional formulations, offering insights into how such combinations can enhance therapeutic outcomes through multi-pathway targeting a perspective not fully covered in many previous studies. This integration of ethnopharmacological knowledge with modern therapeutic strategies underscores the importance of preserving and utilizing traditional medicinal wisdom in contemporary cancer treatment.

### 12.3 Modulation of key oncogenic pathways

Compounds like Brazilin, when combined with standard cancer treatments, show great promise in boosting therapeutic efficacy while minimizing side effects typically associated with chemotherapy and radiotherapy (Khader et al., 2024). These compounds target key oncogenic pathways, such as those regulated by Bcl-2, HER2, p120, MMP-2, and MMP-9—proteins essential for cancer cell survival and metastasis. By modulating these pathways, *C. sappan* not only directly inhibits tumor growth but also sensitizes cancer cells to conventional therapies, enhancing the effectiveness of treatment. Unlike most studies that focus on isolated pathways, our review takes a broader view, highlighting *C. sappan*’s capacity to simultaneously target multiple oncogenic pathways. This multi-target approach is a distinguishing feature of our analysis, offering deeper insights into how these compounds can be integrated into holistic cancer treatments.

### 12.4 Antioxidant, anti-inflammatory, and anti-infectious properties

*C. sappan* exhibits potent therapeutic properties through its antioxidant and anti-infection mechanisms. Its active compounds, such as brazilin and sappanone A, effectively scavenge free radicals, inhibit ROS production, and reduce lipid peroxidation by activating pathways like Nrf2/HO-1, which boost cellular antioxidant defenses, and NF-κB, which suppresses pro-inflammatory mediators. *C. sappan* also protects against oxidative stress-induced apoptosis by activating ERK-MAPK and JAK-STAT pathways, showcasing its potential in managing oxidative stress-related diseases. In addition*, C. sappan* demonstrates strong anti-infection effects by modulating inflammatory and immune pathways. Its compounds inhibit pro-inflammatory mediators like TNF-α, IL-6, and COX-2 via the NF-κB pathway and reduce immune hyperactivation through JAK-STAT modulation, promoting anti-inflammatory cytokines such as IL-10. *C. sappan’*s antibacterial effects stem from disrupting bacterial membrane integrity, while its antiviral properties inhibit viral protein expression and replication. The cumulative evidence underscores the potential of *C. sappan* in effectively managing oxidative stress, combating infections, and alleviating associated inflammatory conditions.

### 12.5 Challenges in transitioning to clinical applications

The potential of *C. sappan* in cancer therapy is highly promising, yet several critical challenges must be addressed to transition from preclinical studies to clinical applications. Key obstacles include regulatory hurdles, safety evaluations, scalability, and the necessity of human trials. Regulatory compliance with stringent safety and efficacy standards demands extensive toxicological evaluations, optimized dosage strategies, and carefully defined therapeutic windows. Current findings, primarily derived from *in vitro* and *in vivo* studies, while insightful, fail to fully replicate the complexity of human physiology and disease progression. This underscores the urgent need for robust clinical trials to validate the efficacy and safety of *C. sappan* in human populations. Additionally, the safe use of natural compounds, including those from *C. sappan*, requires careful oversight. The misconception that natural products are inherently safe often leads to inappropriate usage, particularly without consideration of dose or interactions with other drugs. Effective use of these compounds necessitates a thorough understanding of their mechanisms of action, as well as comprehensive studies across *in vitro*, *in vivo*, and clinical models. Predicted toxicity values for *C. sappan*’s constituents from recent studies indicate low toxicity, suggesting that its bioactive compounds have a favorable safety profile. However, detailed experiments and clinical validation are essential to confirm these findings and ensure their safe application. Another significant challenge lies in scaling up extraction and formulation processes to meet quality control and consistency standards required for clinical use. Furthermore, publication bias, where studies with positive outcomes are disproportionately reported, may create an overly optimistic view of *C. sappan*’s effectiveness. Balanced reporting, including studies with negative or inconclusive results, is crucial to providing an accurate and comprehensive understanding of its therapeutic potential. To fully realize the promise of *C. sappan*, a multi-faceted approach is required. This includes conducting well-structured clinical trials, promoting transparency in research reporting, and addressing issues related to scalability and regulatory compliance. Through these efforts, the therapeutic potential of *C. sappan* can be harnessed to develop safe and effective cancer management strategies, positioning this natural compound as a valuable asset in the fight against cancer.

## 13 Conclusion

The combination of *C. sappan* extracts and isolated compounds with existing cancer treatments presents a promising path for future research. The synergistic interactions observed in traditional formulations and individual compounds suggest the potential of *C. sappan* to enhance the effectiveness of conventional therapies, minimize adverse effects, and provide a more holistic approach to cancer management. Further studies, particularly clinical trials, are essential to fully elucidate the mechanisms and therapeutic benefits of these combination therapies. By emphasizing the plant’s dual role in antioxidant and anticancer mechanisms, and its broader therapeutic benefits spanning cancer, cardiovascular, and metabolic health, this review highlights the extensive potential of *C. sappan* in integrative cancer treatment.
